# An *in vitro* model system based on calcium- and phosphate ion-induced hMSC spheroid mineralization

**DOI:** 10.1016/j.mtbio.2023.100844

**Published:** 2023-11-07

**Authors:** Steven Vermeulen, Kèvin Knoops, Hans Duimel, Maryam Parvizifard, Denis van Beurden, Carmen López-Iglesias, Stefan Giselbrecht, Roman Truckenmüller, Pamela Habibović, Zeinab Tahmasebi Birgani

**Affiliations:** a Department of Instructive Biomaterials Engineering, MERLN Institute for Technology-Inspired Regenerative Medicine, Maastricht University, Maastricht, the Netherlands; b Microscopy CORE Lab, M4I Faculty of Health, Medicine and Life Sciences, Maastricht University, Maastricht, the Netherlands

**Keywords:** Mesenchymal stromal cells, Spheroids, Calcium, Phosphate, Mineralization, Bone, Regenerative medicine

## Abstract

A challenge in regenerative medicine is creating the three-dimensional organic and inorganic *in vitro* microenvironment of bone, which would allow the study of musculoskeletal disorders and the generation of building blocks for bone regeneration. This study presents a microwell-based platform for creating spheroids of human mesenchymal stromal cells, which are then mineralized using ionic calcium and phosphate supplementation. The resulting mineralized spheroids promote an osteogenic gene expression profile through the influence of the spheroids’ biophysical environment and inorganic signaling and require less calcium or phosphate to achieve mineralization compared to a monolayer culture. We found that mineralized spheroids represent an *in vitro* model for studying small molecule perturbations and extracellular mediated calcification. Furthermore, we demonstrate that understanding pathway signaling elicited by the spheroid environment allows mimicking these pathways in traditional monolayer culture, enabling similar rapid mineralization events. In sum, this study demonstrates the rapid generation and employment of a mineralized cell model system for regenerative medicine applications.

## Graphical abstract

**Figure:**
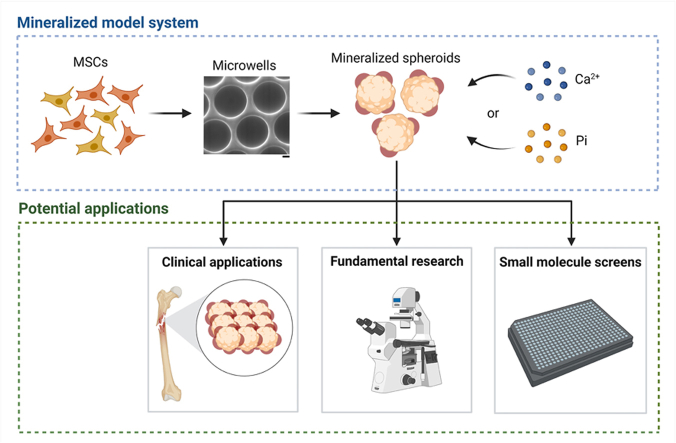

## 1 Introduction

Bone defects caused by traumatic injury or musculoskeletal disorders such as osteoporosis significantly burden individuals and the healthcare system [1]. As a rule, bone has a solid natural capacity to regenerate. Nevertheless, in cases of substantial, critical-sized bone defects or diseased bone tissue, intricate clinical treatments become necessary, often involving prosthetic implants or autologous bone grafts. Despite clinical successes, disadvantages exist, such as a limited lifespan or insufficient osseointegration of prosthetic implants [2,3]. 10.13039/100014337Furthermore, autologous bone grafts, considered the gold standard to support bone healing of large defects due to superior bioactivity and integration [4,5], can only be applied to a limited extent because of their insufficient availability and the risk of donor site morbidity [6]. Therefore, alternative methods are under investigation, such as cell- and biomaterial-based bone-like constructs that resemble tissue physiology, functionality, and durability. However, a challenge in regenerative medicine is recapitulating the complex three-dimensional (3D) bone microenvironment *in vitro*, including its cellular populations and mineralized matrix, which are required to develop novel, successful therapies. Creating such *in vitro* environment would also be helpful as a model system to study developmental processes and disease pathology and aid the discovery of new therapeutics.

Cell aggregation is a promising approach to address this challenge. Cellular assemblies more closely resemble the native tissue physiology than cells cultured in monolayers on flat, two-dimensional (2D) surfaces of conventional culture dishes from polystyrene [7]. This is attributed to signaling events associated with altered cell-cell contacts [8], cell-matrix interactions [9], and cytoskeletal organization [10]. As a result, cellular aggregates have proved to be beneficial for studying developmental processes [11], disease modeling [12], and bottom-up engineering of microtissues for regenerative medicine purposes [13]. Here, mesenchymal stromal cells (MSCs), an easily accessible cell source applicable for tissue repair due to their multipotency [14], present an interesting cell type to be used in the context of cellular aggregation. For example, for obtaining a chondrogenic phenotype, cellular aggregation is a requirement for successful MSC differentiation [15]. Also, in the case of an osteoblastic phenotype, it has been illustrated that aggregation promotes this transition [16]. This association has been proposed to be linked with modifications in key signaling pathways involving altered cadherin [8], bone morphogenetic protein (BMP) [17,18], and WNT signaling [19], crucial pathways involved in osteogenesis [[20], [21], [22]], and supports the notion that cellular aggregation promotes osteogenesis.

Next to these signaling events, calcium (Ca^2+^) and inorganic phosphate (Pi), the main inorganic constituents of bone [23], are not only essential to build bone tissue, but also play crucial signaling roles in maintaining bone physiology [24,25] and osteogenesis *in vitro* [[26], [27], [28], [29]]. For example, adding Ca^2+^ and Pi ions to human MSCs (hMSCs) cultured in a 2D polystyrene dish enhanced osteogenic marker expression and led to rapid mineralization after ten days of culture [30]. While the importance of Ca^2+^ and Pi in bone formation is well-established, their specific roles in spheroid cultures, representing a simplified 3D *in vitro* model, remain largely unknown. Understanding the effects of Ca^2+^ and Pi signaling in the context of spheroid cultures is crucial for advancing our knowledge of bone development and regenerative medicine.

**Fig. 1:**
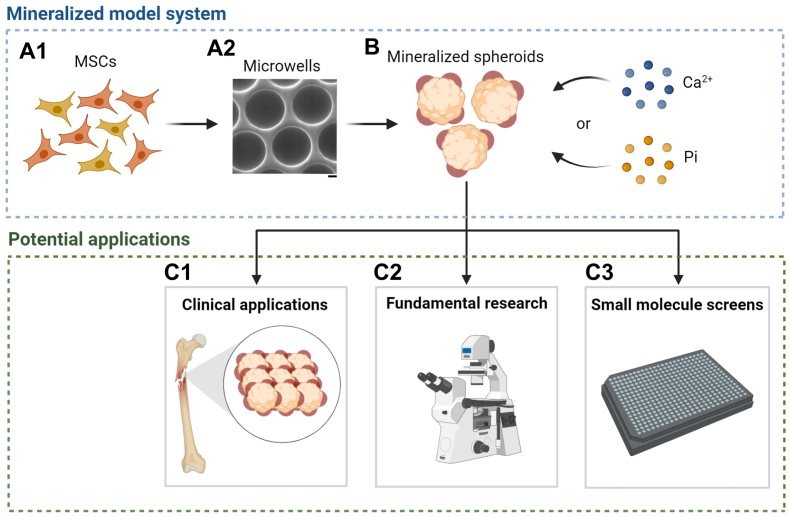
**Schematic overview for developing mineralized MSC spheroid models and its potential applications. A1)** MSCs are used as a cell source to generate mineralized spheroids. **A2)** SEM image of microwell arrays with approximately 500-μm diameter and 300-μm depth that can be inserted in either 24-well or 96-well plates for spheroid generation. The scale bar represents 100 μm. **B)** Spheroid mineralization is achieved by supplementing the culture media with either Ca^2+^ or Pi ions. **C)** Mineralized spheroids are useful for: **C1)** Clinical applications via bottom-up tissue engineering; **C2)** Fundamental research to study developmental processes or biological pathways involving pathological or physiological mineralization; **C3)** Small molecule screens to identify therapeutic drugs that either inhibit or promote mineralization for disease pathologies or regenerative medicine applications.

Therefore, in this work, we explore how adding either Ca^2+^ or Pi affects osteogenic gene expression and mineralization in hMSC spheroids compared to classical osteogenic compounds and the 2D monolayer environment. In the future, these mineralized spheroids may be suitable candidates for implantable/injectable therapeutics for bone regeneration. Furthermore, we demonstrate that spheroid mineralization is a useful model system, which may in the future enable us to study mineralization mechanisms and the identification of novel drug candidates for regenerative medicine applications (Fig. 1). Through these findings, we aim to contribute to the development of innovative therapies for bone-related disorders and advance the field of regenerative medicine.

## 2 Results

### 2.1 Optimization of Ca^2+^ and Pi supplementation for MSC spheroid culture

It is well established that dexamethasone (DEX) and Ca^2+^/Pi supplementation promote osteogenesis in standard culture conditions [26,28,31]. However, the influence of these supplements on osteogenesis in spheroid or organoid constructs is still largely unexplored. In this study, we employed a polymer film-based microwell array setup with a low-binding surface modification to culture bone marrow-derived hMSC (BM-hMSC) spheroids without needing an additional matrix. An advantage is that the interpretation of the role of DEX, Ca^2+^, Pi, and small molecule supplementation can be made without the risk of these compounds being ad- or absorbed by a (hydrogel) matrix [32] (Fig. 1A and **B)**. This setup consists of an array of microwells of approximately 500 μm in diameter and 300 μm in-depth and can easily be integrated into a 24- or 96-well format for cell culture experiments. Promotion of cellular aggregation and inhibition of cell attachment to the polycarbonate (PC) microwells is achieved by Pluronic® F108 coating. Previously, these microwell arrays allowed the generation of intestinal organoids [33], mouse embryonic stem cell aggregates [34], and MSC spheroids carrying microsized biomaterials [35]. Here, we show that seeding MSCs in the microwells rapidly initiates cellular aggregation, with spheroids generated approximately 2 h after cell seeding (Supplementary Data File 1).

**Fig. S1:**
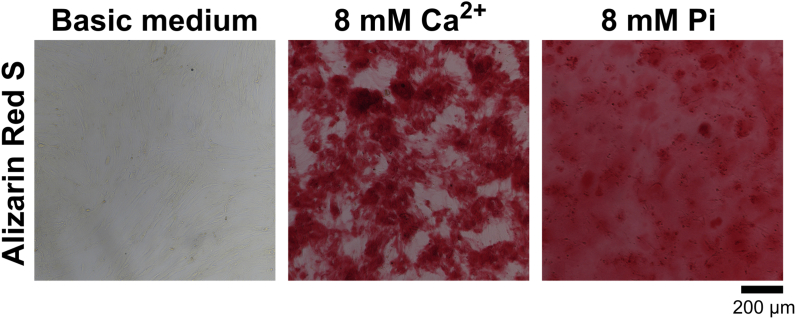
**Supplementary Figure 1:Supplementation the medium with 8 mM Ca^2+^ or 8 mM Pi induces mineralization in a 10-day MSC monolayer culture.** Mineralization stained through Alizarin Red S.

Previously, it was found that the addition of Ca^2+^ or Pi to the culture medium induces mineralization in MSCs 2D cultures after 10 days [30]. To investigate if the MSCs used in this study are able to achieve mineralization under these conditions in 2D monolayers, we added 8 mM Ca^2+^ or 8 mM Pi to the medium and fixated the cells after 10 days of culture. We observed that the addition of these supplements indeed resulted in rapid mineralization after 10 days, as shown by an Alizarin Red S staining, making the MSCs suitable for further experiments (Supplementary Fig. 1).

**Fig. 2:**
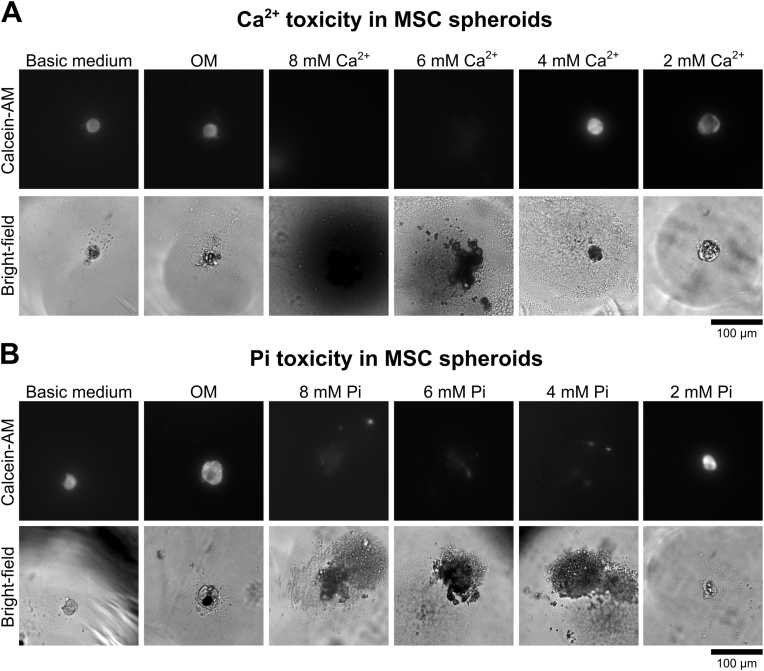
**Ca**^**2+**^**and Pi induce**s **toxicity in MSC spheroids. A)** MSC spheroids incubated in a concentration range of 2–8 mM Ca^2+^ revealed that a concentration higher than 4 mM Ca^2+^ was toxic after 10 days of culture. **B)** MSC spheroids incubated in a concentration range of 2–8 mM Pi indicated that a concentration higher than 2 mM Pi was toxic after 10 days of cell culture. Cell viability was assessed using calcein-AM supplementation. Bright-field images revealed mineral deposits when spheroids were incubated with toxic levels of Ca^2+^ and Pi.

**Fig. S2:**
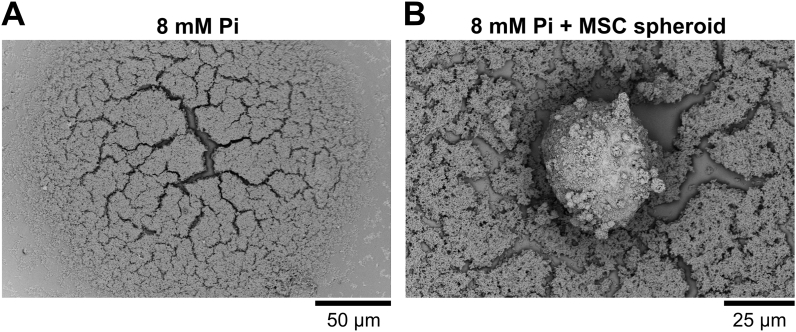
**Supplementary Figure 2: SEM imaging of MSC spheroids incubated for 10 days with 8 mM Pi.A)** Spheroid-free microwells incubated with 8 mM Pi demonstrated mineral deposition. **B)** Spheroids incubated with 8 mM Pi were surrounded by mineral deposition.

Next, we evaluated if Ca^2+^/Pi supplementation leads to toxic effects in MSC spheroids, considering that high Ca^2+^/Pi concentrations are known to impair viability [36,37]. Therefore, we evaluated cell viability in MSC spheroids incubated with 2, 4, 6, and 8 mM Ca^2+^ or Pi by Calcein-AM labeling. After 10 days, little to no viable MSC spheroids were observed in the 6 or 8 mM Ca^2+^ condition, which coincided with a large amount of precipitation in the microwells (Fig. 2A). At 2 and 4 mM Ca^2+^, spheroids remained viable, while for the 4 mM Ca^2+^ condition, the spheroid turned darker, indicating possible mineralization inside the spheroid. Spheroids cultured in standard osteogenic medium (OM) exhibited similar viability as spheroids in basic culture conditions. We observed cell death at concentrations of 4, 6, and 8 mM at 10 days under Pi conditions, which coincided with precipitation (Fig. 2B). At 2 mM Pi, spheroids remained viable while no surrounding deposition was observed. The observed precipitation at high Ca^2+^ and Pi concentrations are likely CaP precipitates resulting from supersaturation of the cell culture medium with Ca^2+^ and Pi, as they were also observed in controlled cell-free conditions (Supplementary Fig. 2A). This precipitation near the spheroid culture is associated with a toxic environment as shown by the absence of viable cells. Scanning electron microscopy (SEM) imaging further confirmed that spheroids are exposed to severe precipitation at high Ca^2+^ and Pi levels (Supplementary Fig. 2B). These results demonstrate that choosing correct Ca^2+^ and Pi supplementation in the culture medium is an important consideration for MSC spheroid viability.

### 2.2 Ca^2+^ and Pi supplementation induces rapid mineralization in MSC spheroids

**Fig. 3:**
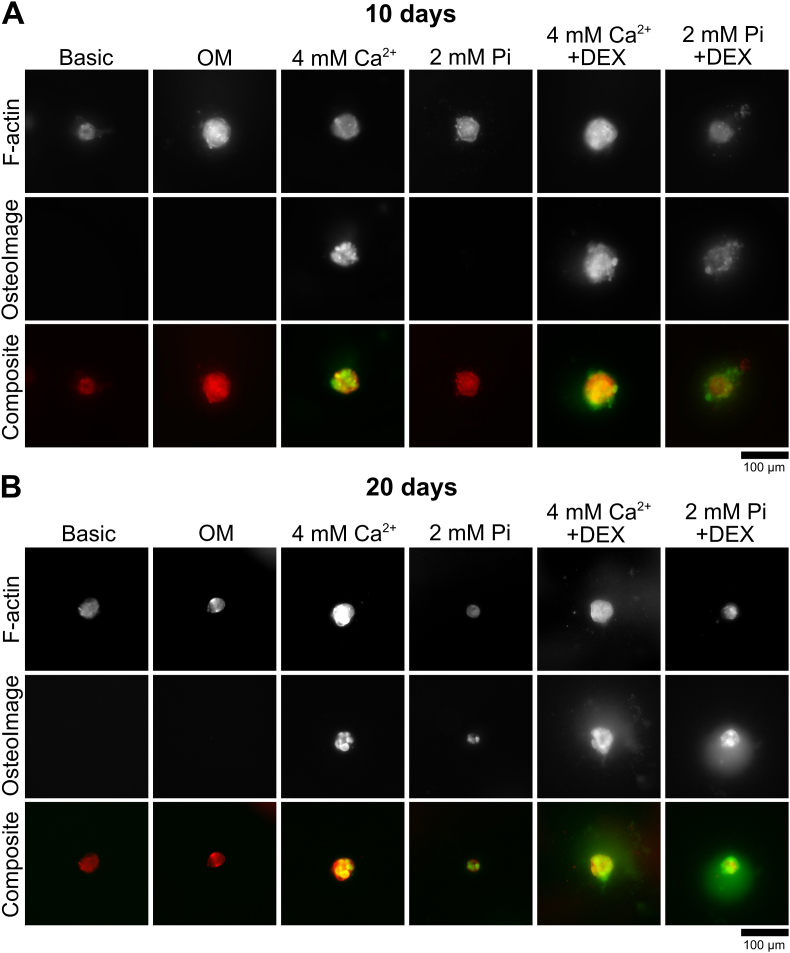
**Impact of Ca**^**2+**^**, Pi, and DEX supplementation on MSC spheroid mineralization. A)** After 10 days, 4 mM Ca^2+^ induced mineralization in MSC spheroids. 2 mM Pi induced mineralization only when DEX was supplemented. After 10 days, no mineralization was visible in the OM condition. **B)** After 20 days, mineralization increased in all conditions except for spheroids cultured in basic medium and OM. Low mineralization levels were observed in the 2 mM Pi condition, while DEX supplementation further elevated mineralization in the Pi condition. Spheroids were stained for F-actin through Phalloidin-568 (red). Mineral deposition was stained using OsteoImage™ dye (green). OM = Osteogenic medium.

**Fig. S3:**
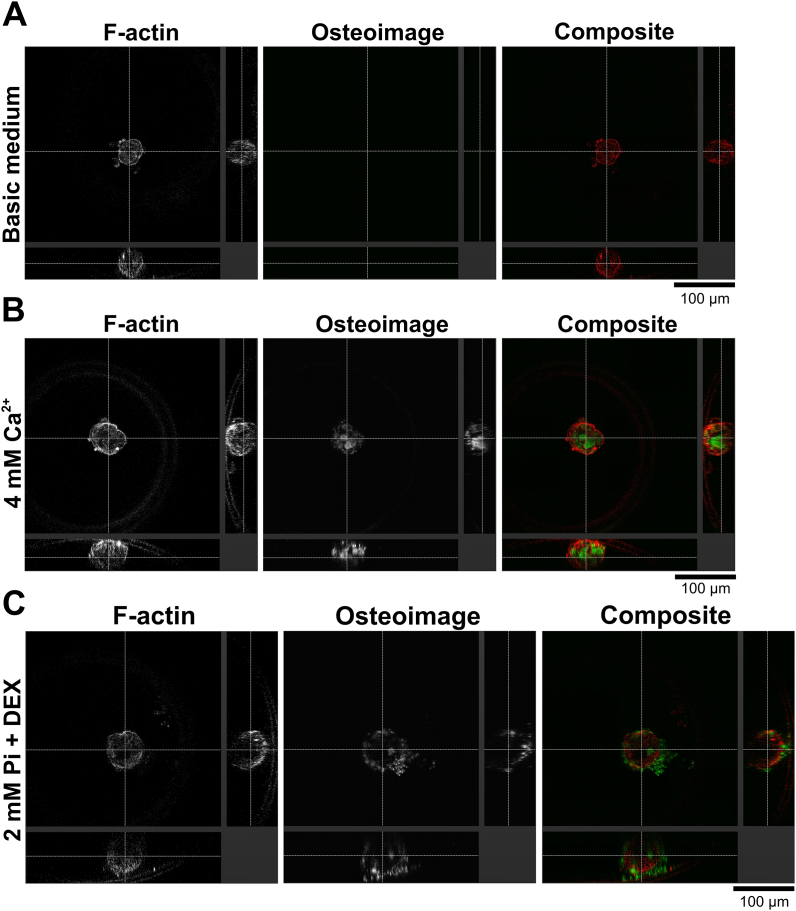
**Supplementary Figure 3: Confocal images and orthogonal sections of MSC spheroids in basic medium and treated with either 4 mM Ca^2+^ or 2 mM Pi + DEX for 10 days.A)** MSC spheroids in basic conditions did not demonstrate mineralization. **B)** Mineral formation inside spheroids occured when incubated with 4 mM Ca^2+^. **C)** Both internal and external mineralization were present in spheroids incubated with 2 mM Pi + DEX. F-actin was stained with Phalloidin (red) and hydroxyapatite with the OsteoImage™ dye (green).

**Fig. S4:**
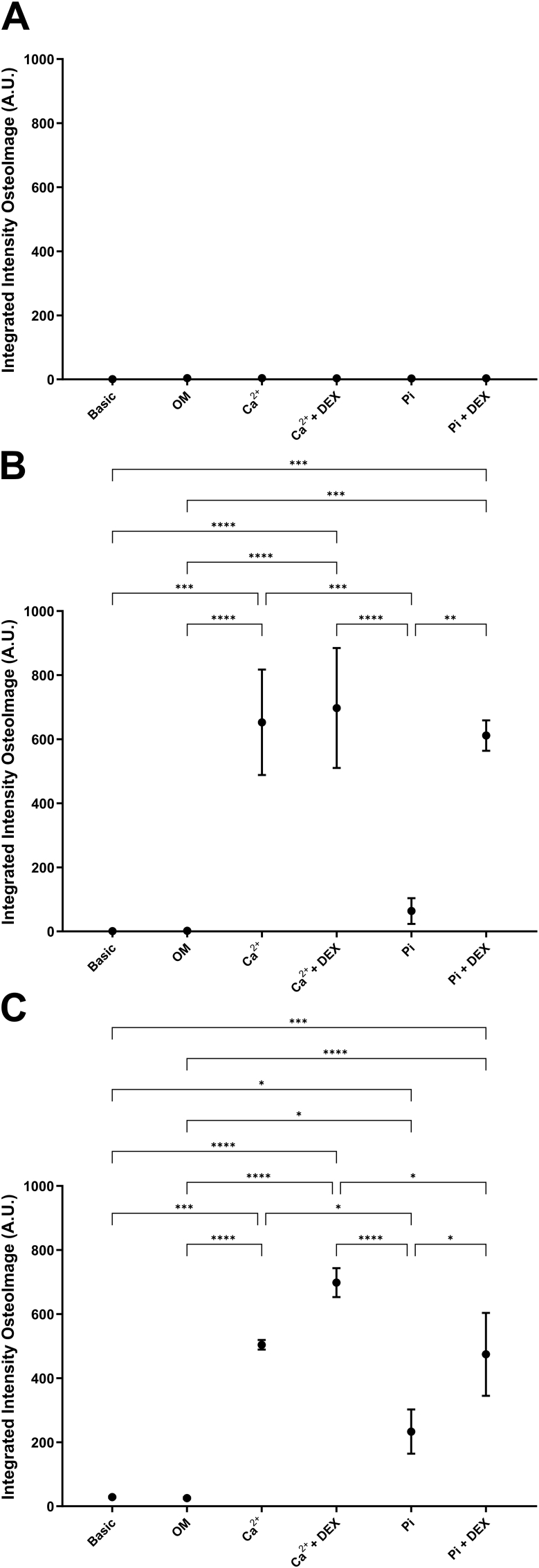
**Supplementary Figure 4: Quantification of spheroid mineralization levels. A)** At day 5, no visible mineralization was detected. **B)** At day 10, mineralized deposits were detectable in the Ca^2+^, Ca^2+^ + DEX and Pi + DEX condition. Very low to no mineralization was detected in the Pi condition. **C)** At day 20, mineralized deposits were detectable in all conditions. Each dot represent the mean of osteoimage levels detected in multiple spheroids. Error bars represent SEM (*P < 0.05; ** P < 0.01; *** P < 0.001; **** P < 0.0001).

**Fig. S5:**
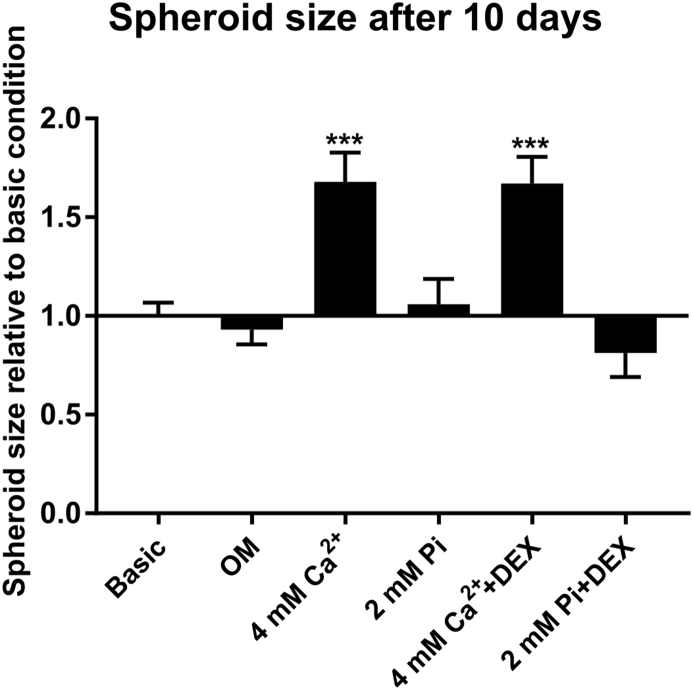
**Supplementary Figure 5: Spheroids treated with Ca^2+^ exhibit an approximately 50% increase in size compared to spheroids cultured in basic medium.** F-actin was stained with Phalloidin (red) and hydroxyapatite with the OsteoImage™ dye (green) (*** P < 0.001). Error bars represent SEM.

**Fig. S6:**
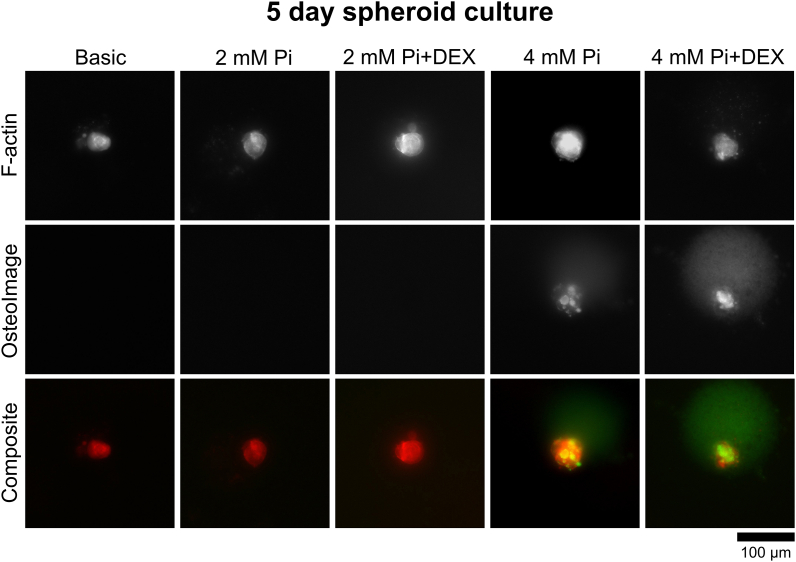
**Supplementary Figure 6: Treating MSC spheroids with 4 mM Pi and DEX induces mineralization after 5 days.** Similar to the mineralization observed after 10 days of incubation with 2 mM Pi, DEX further enhanced deposition. At 4 mM Pi, non-biological mineralized deposition surrounding the spheroids was visible. F-actin was stained with Phalloidin (red) and hydroxyapatite with OsteoImage™ (green).

**Fig. S7:**
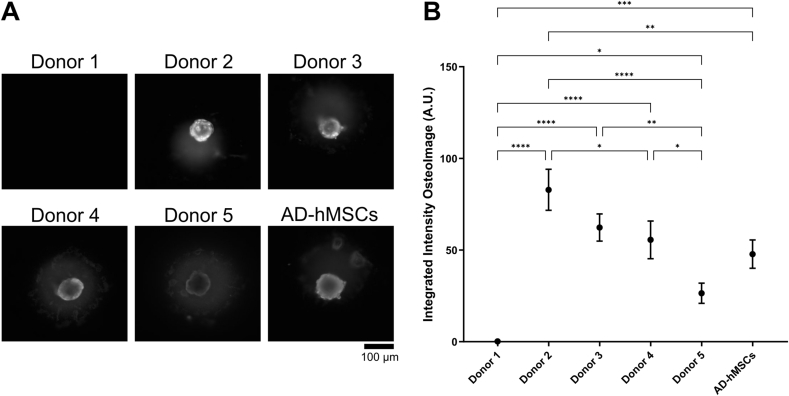
**Supplementary Figure 7: Mineralization variability across different BM-hMSC donors and AD-hMSCs.** **A)** Five different BM-hMSC donors and one AD-hMSC donor exhibited different calcification potential when subjected to 4 mM Ca^2+^ supplementation. Donor 1 did not calcify under these conditions, while donor 5 exhibited a low potential. **B)** Quantification of OsteoImage intensity confirmed these observations (*P < 0.05; ** P < 0.01; *** P < 0.001). Error bars represent SEM.

Since the data obtained so far suggests that spheroids remained viable at 4 mM Ca^2+^ and 2 mM Pi, we investigated if spheroid mineralization is induced at these concentrations. For this, we stained spheroids with OsteoImage™, a fluorescent dye that binds to calcium phosphate [38]. We further labeled cytoskeletal F-actin with Phalloidin for spheroid visualization. On day 5, mineralization was not visible at these concentrations (data not shown). On day 10, mineralization was present in the Ca^2+^ condition, yet not in the 2 mM Pi condition (Fig. 3A). DEX in combination with Pi however supported mineralization in spheroids, similar as seen in regular 2D culture conditions. Confocal imaging confirmed these observations (Supplementary Fig. 3). When mineralization occurred, we could occasionally detect mineralization outside the spheroids as shown for the 2 mM Pi + DEX condition. At 20 days, mineralization further increased in the 4 mM Ca^2+^ condition without an observable difference when DEX was included (Fig. 3B). We continued to see a build-up of minerals for the Pi condition compared to the 10-day time-point and the enhancing effect of DEX. These particular MSCs did not exhibit mineralization with OM after 20 days. Quantification of the OsteoImage™ intensity levels confirmed these observations (Supplementary Fig. 4). Mineralized deposition outside the spheroids continued to be observed as shown for the 4 mM Ca^2+^ + DEX and 2 mM Pi + DEX condition. Interestingly, spheroids treated with Ca^2+^ (+DEX) increased in size, likely due to mineral or matrix formation. Quantification of spheroid size using epifluorescence images showed that this effect manifested as early as 10 days. Both the 4 mM Ca^2+^ and 4 mM Ca^2+^ + DEX conditions expanded the spheroid size by around 50 % relative to the basic medium conditions (Supplementary Fig. 5). We observed that spheroids maintained under basic conditions exhibited no further increase in size, possibly suggesting a lack of cell proliferation in these spheroids. In a previous study, we demonstrated that MSCs cultured in spheroids lose their proliferation potential [35], an observation confirmed by other researchers [39,40]. We further investigated if the ion concentrations affected mineralization speed. Although adding more than 2 mM Pi in the medium is toxic for the spheroids after 10 days, mineralization was observed already after 5 days when spheroids were treated with 4 mM Pi or 4 mM Pi + DEX (Supplementary Fig. 6). Previous research indicated that donor variability influences calcification potential of MSCs in 2D conditions [30]. We therefore wondered if MSC donor variability affects mineralization in spheroid conditions as well. Hence, we investigated mineralization in BM-MSC from multiple donors, and included adipose-derived MSCs (AD-MSCs) as well to evaluate if other MSC cell sources can be utilized in our experimental setup. Mineralization potential varied between the five BM-MSC donors, with one donor exhibiting no mineralization, one donor exhibiting low mineralization potential, and three donors exhibiting higher mineralization potential compared to the previous two donors (Supplementary Fig. 7). The AD-MSC donor exhibited a similar potential compared to the high performing BM-MSC donors. These findings suggest that choosing the dosage of Ca^2+^ or Pi supplementation, as well as the cell source, is important to avoid toxic levels while achieving rapid mineralization in spheroids.

**Fig. 4:**
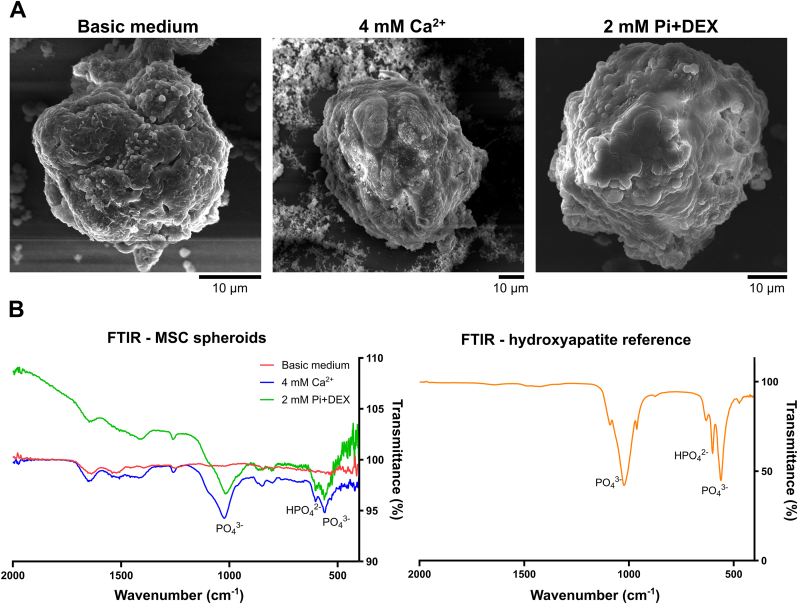
**FTIR analysis indicates the presence of hydroxyapatite in mineralized MSC spheroids. A)** SEM images of MSC spheroids cultured in basic medium, 4 mM Ca^2+^ and 2 mM Pi + DEX. **B)** FTIR spectra from the spheroids' dry mass displayed prominent PO_4_^3−^ peaks when spheroids are treated with calcification medium, which corresponds to the spectrum of the hydroxyapatite reference sample.

**Fig. S8:**
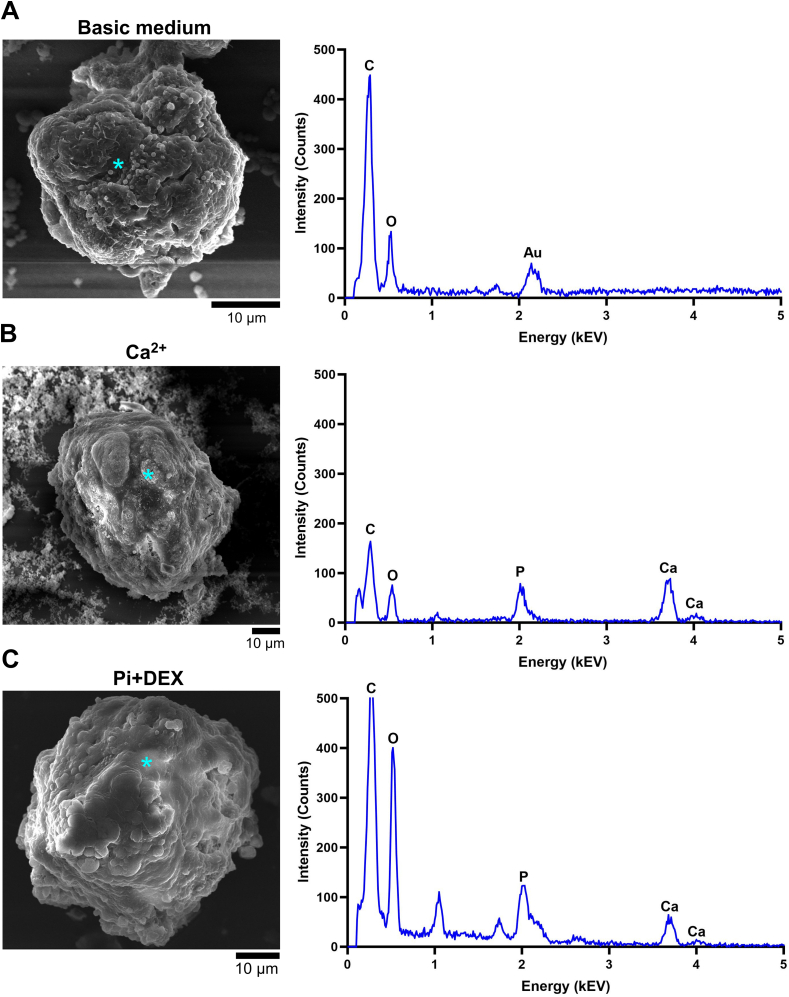
**Supplementary Figure 8: EDS spectral analysis of spheroid cultures. A)** Spheroids cultured in basic medium show an absence of detectable Ca and P on their surface. **B)-C)** In contrast, spheroids grown in medium containing 4 mM Ca^2+^ or 2 mM Pi + DEX demonstrate surface accumulation of Ca and P. The blue asterisk indicates the location of the recorded EDS spectra.

To elucidate the nature of this mineralization, we employed SEM with energy-dispersive X-ray spectroscopy (EDS) for elemental composition analysis. Spheroids exposed to calcification medium containing 4 mM Ca^2+^ or 2 mM Pi + DEX (Fig. 4A) exhibited a presence of minerals, which was particularly visible in the 4 mM Ca^2+^ condition, where EDS spectra pinpointed an elevated amounts of Ca and P elements on these spheroid surfaces (Supplementary Fig. 8). Additionally, using Fourier-transform infrared spectroscopy (FTIR) on the spheroids' dry mass helped identifying the chemical composition of the deposited minerals. Spheroids treated with 4 mM Ca^2+^ and 2 mM Pi + DEX exhibited pronounced PO_4_^3−^ peaks, mirroring a hydroxyapatite reference sample (Fig. 4B).

**Fig. 5:**
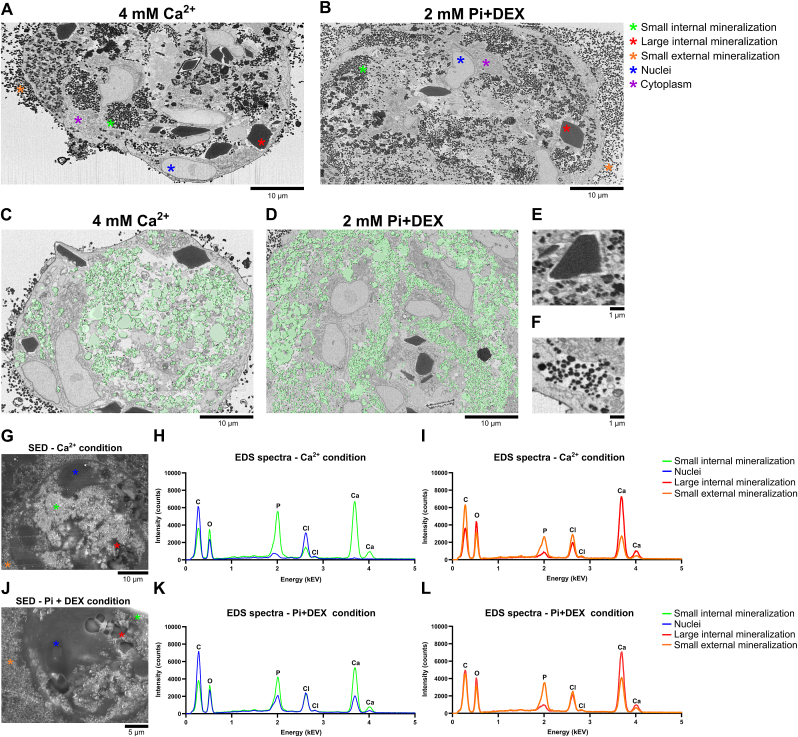
**Characterization of mineralized deposits in MSC spheroids via FIB-SEM imaging and EDS analysis. A)** MSC spheroids incubated with 4 mM Ca^2+^ for 10 days exhibited small and large mineral deposits predominantly located inside the spheroids. **B)** Spheroids incubated with 2 mM Pi + DEX for 10 days similarly exhibited small and large mineral deposits. Smaller mineral deposits were also strongly present outside the spheroids. **C** and **D)** Segmentation of the smaller mineral deposits inside the spheroids demonstrated that mineralization was present throughout the spheroids, yet not in the nuclei, for both the 4 mM Ca^2+^ and 2 mM Pi + DEX conditions. **E** and **F)** Closer inspection revealed that the smaller and larger mineral deposits were located outside the cells and between the cell membranes. **G)** SED visualization of an MSC spheroid treated with 4 mM Ca^2+^. **H)** EDS spectra analysis revealed that the smaller mineral deposits exhibited a strong presence of Ca and P in the 4 mM Ca^2+^ condition. **I)** Elemental spectra analysis revealed that the larger mineral deposits exhibited a high presence of Ca and a low presence of P. **J)** SED visualization of an MSC spheroid treated with 2 mM Pi + DEX. **K)** Smaller mineral deposits exhibited a strong presence of Ca and P in the 2 mM Pi + DEX condition. **L)** Larger mineral deposits in the 2 mM Pi + DEX condition exhibited a high presence of Ca and a low presence of P.

**Fig. S9:**
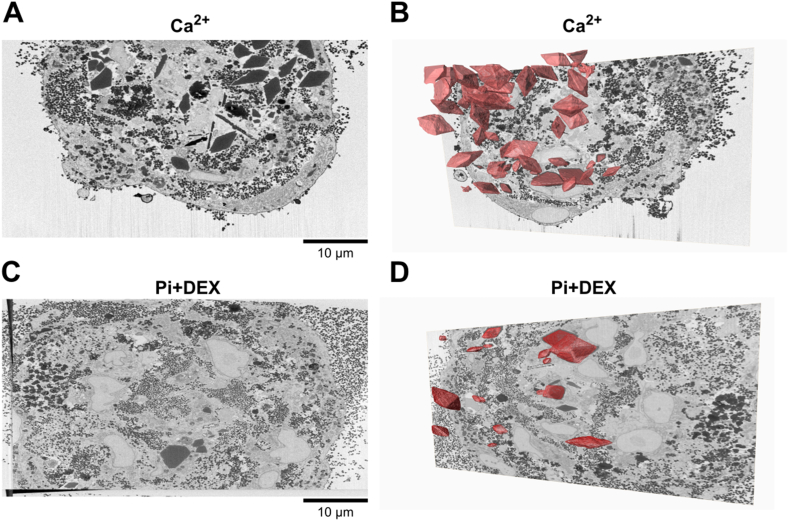
**Supplementary Figure 9:** 3D rendering of the large octahedron-shape minerals in 4 mM Ca^2+^ and 2 mM Pi + DEX conditions.

To investigate these minerals in closer detail and greater accuracy we applied focused ion beam-SEM (FIB-SEM) imaging to allow the visualization of the mineral deposits inside the spheroids as well. Similarly, we also applied EDS to investigate their elemental composition inside and outside the spheroids. FIB-SEM allowed the visualization of stacks of micrometer thin slices inside the spheroids and enabled generating a 3D reconstruction of the spheroids (Supplementary Data Files 2–4). FIB-SEM applied to spheroids treated with 4 mM Ca^2+^ or 2 mM Pi + DEX for 10 days revealed small dot-like minerals inside and outside the spheroids and large octahedral minerals inside the spheroids (Fig. 5A and B). The 3D rendering of the spheroids revealed that these large octahedron-shaped structures were more frequently observed in the 4 mM Ca^2+^ condition compared to the 2 mM Pi + DEX condition (Supplementary Data Files 2–4 and Supplementary Fig. 9). Consistent with the findings from OsteoImage™ stainings, deposits were evident outside the spheroids in the 2 mM Pi + DEX condition and the 4 mM Ca^2+^ condition, though to a lesser extent in the latter. Segmentation, outlined in green, of the smaller mineral deposits, further illustrates that mineralization was strongly present inside the spheroids (Fig. 5C and D). Mineralization did not seem to occur intracellularly, as shown in Fig. 5E and F, where intercellular space, illustrated by a light-grey background, surrounded the smaller and larger mineral deposits.

**Fig. S10:**
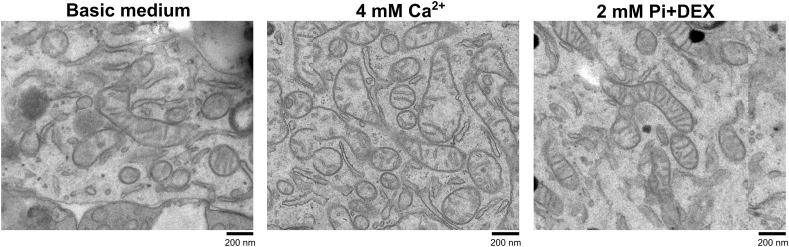
**Supplementary Figure 10:** TEM imaging shows consistent mitochondrial morphology across all conditions.

To investigate if mineralization affected subcellular components, we utilized transmission electron microscopy (TEM). We observed no differences across the test conditions. Notably, the mitochondria maintained their typical morphological features with no discernible size differences between spheroids cultured in basic medium, 4 mM Ca^2+^, and 2 mM Pi + DEX (Supplementary Fig. 10). Considering that an increase of mitochondrial fusion can be indicative of cellular stress [41], this observation supports the earlier observation that cell viability remains unaffected between the basic medium condition and conditions with 4 mM Ca^2+^ or 2 mM Pi + DEX supplementation.

EDS elemental analysis on various spheroid locations, visualized by the secondary electron detector (SED) (Fig. 5G), demonstrated a high presence of Ca and P elements in the small minerals compared to the nuclei (Fig. 5H). Ca and P peaks of mineralization outside the spheroids are lower in intensity than internal mineralization, likely due to these minerals being less dense (Fig. 5I). Of interest, the larger minerals exhibited a substantial increase in Ca, while P was nearly absent. This indicates that these minerals have a different chemical composition than the smaller minerals. Visualization of the spheroid by SED in the 2 mM Pi + DEX condition showed a similar distribution of the smaller elements as the 4 mM Ca^2+^ condition, although here outside mineralization was more prominent (Fig. 5J). The EDS spectra analysis revealed that, similar to the 4 mM Ca^2+^ condition, both Ca and P elements were present in the small minerals inside and outside the spheroid (Fig. 5K and L). By measuring the stoichiometric ratio of Ca/P of the small minerals present inside and outside the spheroids, we found that these closely resemble a ratio of 1.67, the Ca/P ratio of hydroxyapatite, thereby highly suggesting that these are indeed hydroxyapatite minerals (Table 1). Similar elemental spectra for the larger minerals were observed as those in the 4 mM Ca^2+^ condition (Fig. 5L). We suggest that these minerals are calcium oxalate, known to be present in fetal bovine serum [42]. The octahedral structure typical for calcium oxalate minerals, further supports the notion that these are calcium oxalate crystals [43]. It might be possible that the addition of Ca^2+^ increased the precipitation of calcium oxalate from the cell culture medium [44], thereby increasing their presence inside the spheroid due to absorption. These results demonstrate that the smaller minerals inside and outside the spheroids exhibit a Ca/P profile similar to hydroxyapatite, while the spheroids also take up calcium oxalate minerals from the culture medium.

**Table 1:** Atomic Ca/P ratio for minerals inside and outside the spheroids.

Condition	Location	Atomic Ca/P ratio
4 mM Ca^2+^	Internal mineralization - small minerals	1.67
	External mineralization	1.62
2 mM Pi + DEX	Internal mineralization - small minerals	1.61
	External mineralization	1.61

### 2.3 The spheroid environment, inorganic factors, and DEX signaling influence osteogenic gene expression

**Fig. 6:**
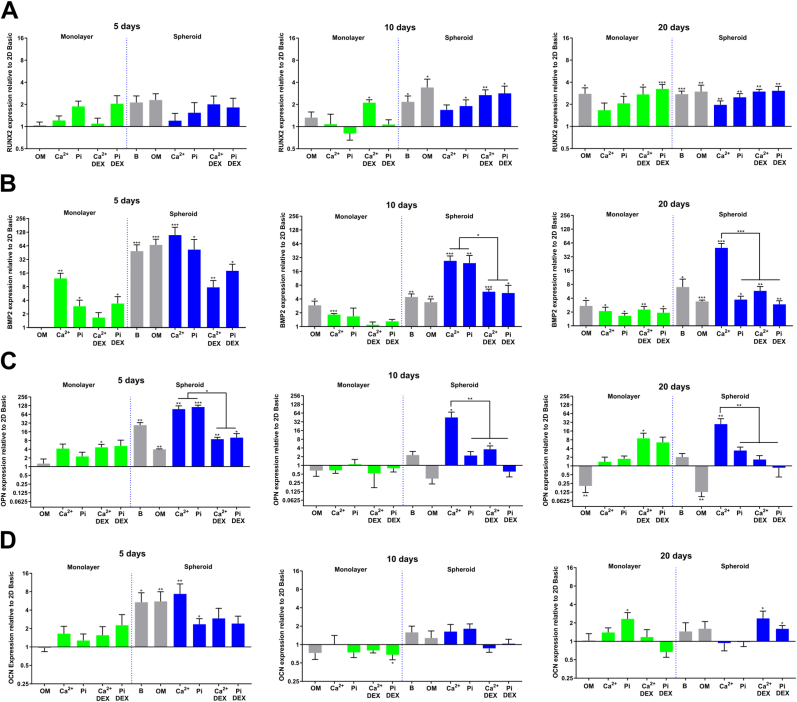
**Osteogenic marker expression in response to a spheroid** e**nvironment and Ca**^**2+**^**, Pi, and DEX supplementation. A)** RUNX2 expression progressively increased for most conditions, reaching its peak after 20 days. Spheroid conditions induced an early elevation of RUNX2 levels comparable to a 20-day incubation with OM or Ca^2+^/Pi (+DEX)) in monolayer conditions. **B)** BMP2 is upregulated early by Ca^2+^/Pi supplementation and the spheroid environment, while synergistic effects are observed when Ca^2+^/Pi and spheroid conditions are combined. DEX decreases BMP2 expression in spheroids. **C)** OPN followed a similar pattern as BMP2, with synergistic effects present when Ca^2+^ and spheroid conditions are combined. **D)** OCN exhibited the highest expression levels at 5 days in a spheroid environment. Afterward, OCN levels drop, with only a significant upregulation found in monolayers treated with Pi and in spheroid conditions with Ca^2+^/Pi supplemented with DEX after 20 days. mRNA levels were determined by qRT-PCR and expression levels are relative to an MSC monolayer cultured in basic conditions of the same time point (*P < 0.05; **P < 0.01; ***P < 0.001).

**Fig. 7:**
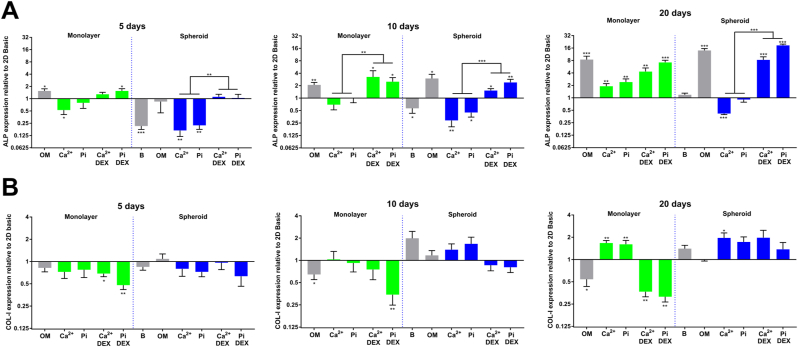
**Expression of the osteogenic markers ALP and COL-I are mainly affected by DEX and inorganic signaling. A)** ALP levels were increased by DEX supplementation across all time points and in both monolayer and spheroids conditions. Spheroid conditions led to reduced ALP levels, especially at early time points. **B)** COL-I expression levels in monolayer conditions were downregulated by DEX supplementation. COL-I mainly exhibited increased expression levels in spheroid conditions after 20 days of culture. mRNA levels were determined by qRT-PCR and expression levels are relative to an MSC monolayer cultured in basic conditions of the same time point (*P < 0.05; **P < 0.01; ***P < 0.001).

To evaluate the phenotypical effects of Ca^2+^, Pi, and DEX supplementation in both MSC spheroids and monolayer cultures, we evaluated the expression of the osteogenic markers bone morphogenetic protein 2 (BMP2), osteopontin (OPN), osteocalcin (OCN), RUNX family transcription factor 2 (RUNX2), alkaline phosphatase (ALP), and collagen-I (COL-I), at 5, 10, and 20 days. In general, we found that both Ca^2+^, Pi, and the spheroid environment increased BMP2, OPN, OCN, and RUNX2 levels (Fig. 6). In contrast, ALP and COL-I were mainly influenced by DEX (Fig. 7).

For RUNX2, an essential osteogenic transcription factor [45], we observed higher yet borderline significance levels (P < 0.1) at 5 days, especially for the Pi-treated monolayer culture and spheroids in basic medium and OM (Fig. 6A). After 10 days, statistically significant higher levels were present in all spheroid conditions, except for a borderline significance (P < 0.1) when spheroids were treated with Ca^2+^. Only cells treated with Ca^2+^ + DEX reached significant levels in the monolayer conditions. After 20 days, the monolayer cultures reached statistically significant levels for all conditions except for the Ca^2+^ treatment. Of interest, spheroids cultured in basic medium obtained similar, yet earlier expression levels as various monolayer conditions at later time points. Throughout the observed time points, DEX slightly increased RUNX2 expression in both monolayer and spheroid conditions. As expected, RUNX2 followed a steady increase over time when applying classic OM in a monolayer culture. These results demonstrate that a spheroid environment can facilitate osteogenic differentiation by an earlier upregulation of RUNX2 compared to a monolayer culture.

BMP2, a crucial growth factor for maintaining bone homeostasis and osteogenesis [46], was upregulated when MSCs in a monolayer were exposed to Ca^2+^ or Pi compared to basic culture medium (Fig. 6B), which is in line with observations made by other groups [26,30]. Similarly, as demonstrated by others, MSC spheroids in basic culture medium showed remarkably higher BMP2 levels compared to a monolayer culture in basic culture medium [17,18]. Of interest is that BMP2 levels gradually decreased over time in most conditions. In this regard, the spheroid environment and Ca^2+^/Pi supplementation induced a synergistic effect on BMP2 levels compared to the Ca^2+^/Pi supplementation in a monolayer culture and spheroids cultured in basic medium, which was most evident at 10 days. After 20 days, only spheroids treated with Ca^2+^ continued to exhibit this effect. DEX had a detrimental effect on BMP2 expression levels in spheroids at all time points, while no influence was observed in the monolayer culture after 20 days, demonstrating that Ca^2+^ or Pi, but not DEX, is a major contributor to BMP2 expression.

For OPN, a prominent bone matrix protein [47], we observed that expression levels of MSC spheroids followed a similar pattern as BMP2 (Fig. 6C). After 5 days, a synergistic effect on OPN expression induced by the spheroid environment and Ca^2+^/Pi was observed compared to a spheroid environment in basic conditions and the Ca^2+^/Pi supplementation in the monolayer culture. In contrast to BMP2, treating MSC spheroids with Pi induced a synergistic effect only at the 5-day time point, while for Ca^2+^, this effect lasted for 20 days. Similarly, as shown for BMP2, DEX diminished the expression levels of the spheroids to levels comparable to the monolayer culture. In contrast, DEX increased expression levels in the Ca^2+^ condition of the monolayer culture after 20 days, demonstrating that the effect of soluble cues can be different in spheroid culture. After 10 days, OPN levels decreased considerably, with only a significant elevation detected in spheroids treated with Ca^2+^. These levels persisted for 20 days, while the monolayer culture treated with Ca^2+^ and DEX also exhibited elevated levels. Interestingly, after 20 days of OM treatment, OPN levels decreased considerably in both the monolayer cultures and spheroids.

For OCN, a bone matrix protein and osteogenic marker [48], we observed substantial upregulation at 5 days compared to later time points (Fig. 6D). Here, the spheroid culture in basic medium conditions was sufficient to induce a strong upregulation, while Pi and DEX lowered OCN levels in spheroids. In contrast, after 10 days, no statistically significant differences were observed between the conditions. After 20 days, statistically significant expression levels were only detected for the monolayer treated with Pi and the Ca^2+^ + DEX and Pi + DEX-treated spheroids. Although OCN is regarded as a late osteogenic marker, a possible explanation for the higher expression levels at 5 days is that the early mineralization in spheroids triggers OCN expression due to its role in bone remodeling through interaction with hydroxyapatite [49].

For ALP, a phosphatase that promotes bone mineralization [50], the expression levels were positively modulated by DEX, while lower ALP expression was observed after 5 and 10 days in the spheroid conditions (Fig. 7A). In general, ALP expression was highest after 20 days of culture. At this time point, ALP expression in spheroids treated with OM, and Ca^2+^/Pi with DEX, also reached similar levels as in the monolayer culture. Furthermore, Ca^2+^/Pi without DEX supplementation in a monolayer culture increased ALP expression only after 20 days. Since ALP is strongly involved in mineralization, it might be plausible that DEX promoted the mineralization events observed in the 2 mM Pi + DEX condition despite DEX decreasing BMP2 and OPN levels.

For COL-I expression levels, the main protein component of the bone matrix, we observed that DEX decreased COL-I expression levels in the monolayer culture but minimally in the spheroid conditions (Fig. 6B). After 20 days, Ca^2+^/Pi treatment increased COL-I expression levels in monolayer and spheroid conditions, yet with only a borderline statistical significance (P < 0.1) for spheroids treated with Pi. The above results demonstrate how distinct biophysical, inorganic, and biochemical cues converge upon the osteogenic gene expression profile in MSCs.

### 2.4 Mineralized MSC spheroids exhibit a long-term osteogenic profile

**Fig. 8:**
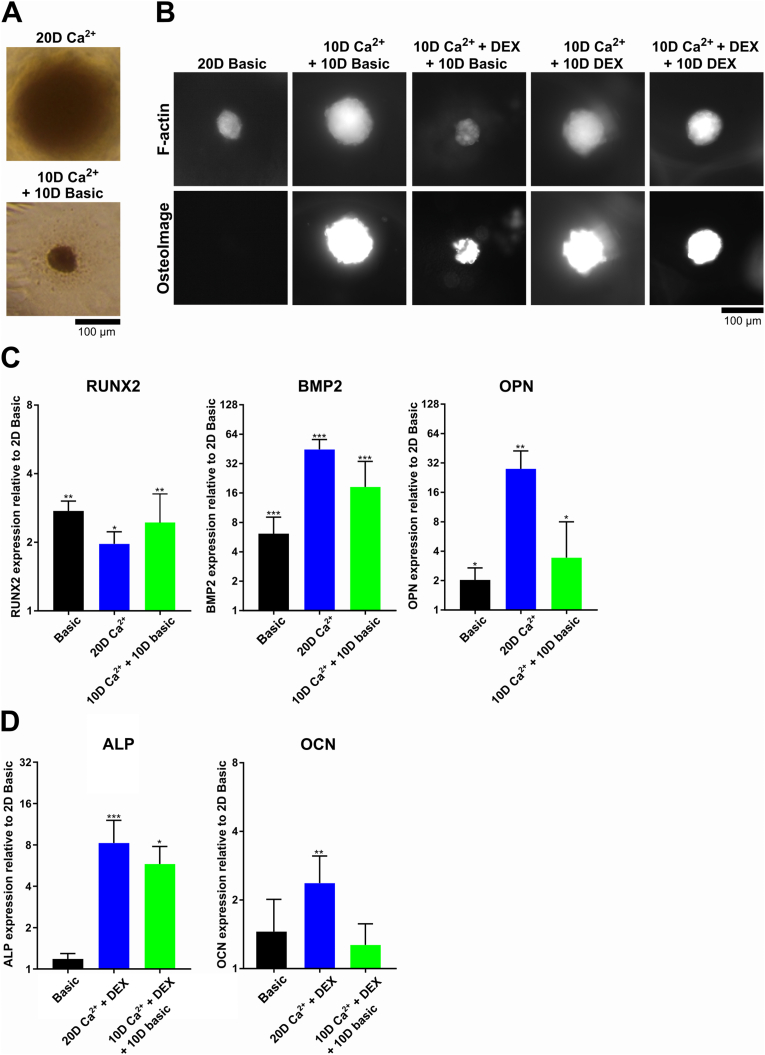
**Spheroid mineralization remains stable after switching in basic culture conditions and supports the expression of the osteogenic markers RUNX2, BMP2, and ALP**. **A)** After 20 days of exposure to 4 mM Ca^2+^, mineralized deposition surrounding the spheroids- was visible. This phenomenon is not observed when the medium is reverted to basic medium after 10 days. **B)** Regardless of the transition back to the basic medium or a medium supplemented with DEX, mineralization continued to be visible after 20 days. **C**) BMP2 and RUNX2 expression levels drop slightly or are unaffected when spheroids were switched back to basic medium. OPN reached similar expression levels as spheroids treated for 20 days with basic medium. **D)** ALP expression after switching back to basic media is at slightly decreased levels compared to 20 days of continuous Ca^2+^ supplementation with DEX. OCN reached similar expression levels as spheroids treated for 20 days with basic medium. mRNA levels were determined by qRT-PCR and all expression levels are relative to an MSC monolayer cultured in basic conditions of the same time point (*P < 0.05; **P < 0.01; ***P < 0.001). 10D = 10 days; 20D = 20 days.

Having established that supplementation with Ca^2+^/Pi triggers rapid mineralization in MSC spheroids, we wanted to explore whether this mineralization alone could sustain an osteogenic gene expression signature, without further need for Ca^2+^/Pi supplementation. This is relevant for bone tissue engineering applications, such as aggregating multiple spheroids (Fig. 1C) for subsequent implantation, as shown by others [13]. Another consideration is that although spheroids remain viable after 20 days of 4 mM Ca^2+^ incubation, we observed mineralized deposition outside the spheroids similar to earlier time points at higher concentrations, which might result in harmful conditions in extended culture times. Therefore, we cultured the spheroids back in basic medium after a 10-day incubation with 4 mM Ca^2+^ and found that we could avoid this phenomenon (Fig. 8A). Of interest, OsteoImage™ staining revealed that switching to basic medium (+DEX) after a 10-day 4 mM Ca^2+^ (+DEX) incubation did not decrease the mineralized content inside the spheroids (Fig. 8B). In addition, including DEX in the basic medium after a 10-day 4 mM Ca^2+^ (+DEX) incubation did not seem to have a further beneficial effect on mineralization.

Next, we aimed to establish if these conditions had similar gene expression profiles as a 20-day incubation with 4 mM Ca^2+^ (+DEX). For this, we targeted the genes RUNX2, BMP2, ALP, OPN, and OCN and selected a 20-day incubation and 10-day incubation with either 4 mM Ca^2+^ or 4 mM Ca^2+^ + DEX, depending on which condition elevated these targets. For RUNX2, BMP2, and OPN we chose the 4 mM Ca^2+^ condition (Fig. 8C) since DEX inhibited BMP2 and OPN levels and had minimal effects on RUNX2. For ALP and OCN, we chose the 4 mM Ca^2+^ + DEX condition (Fig. 8D) since DEX further stimulated these expression levels. BMP2 levels slightly decreased in the 10-day 4 mM Ca^2+^ + 10-day basic medium condition yet remained at higher levels than MSC spheroids cultured in basic conditions. RUNX2 levels of MSC spheroids treated with 4 mM Ca^2+^ (+DEX) were not affected when medium was changed to basic medium after 10 days, while ALP levels slightly decreased. Only for OPN and OCN we detected a decrease in expression levels towards similar levels as spheroids incubated in basic medium for 20 days. These observations suggest that continuous Ca^2+^ addition elevates OPN and OCN levels, but the mineralized deposits alone are inadequate to maintain this elevation. This observation is intriguing, given that OPN and OCN play key roles in facilitating mineral formation [49,51] and thus might not remain upregulated in an already mineralized environment where additional mineralization through Ca^2+^ supplementation is not stimulated.

### 2.5 Spheroid mineralization as a model system to study small molecule perturbations and extracellular deposition

**Fig. 9:**
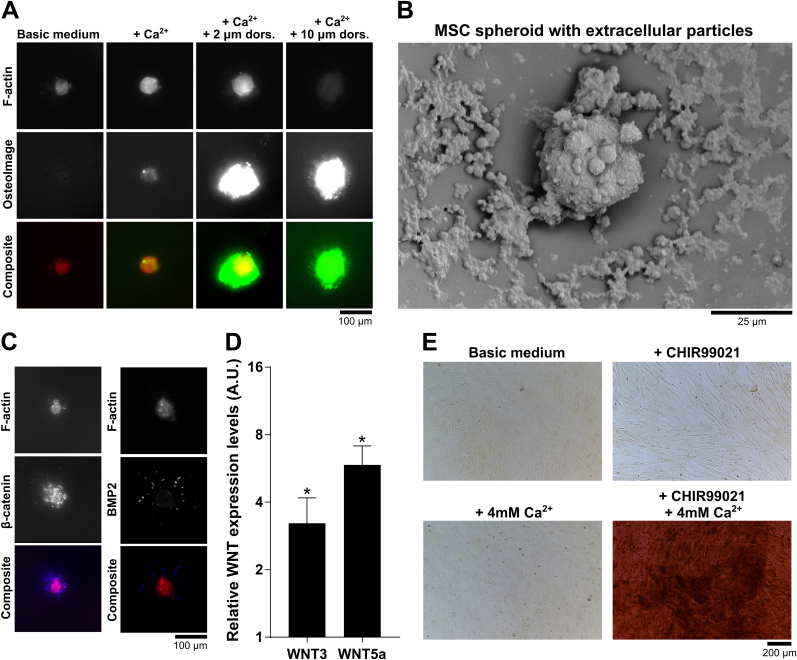
**Rapid spheroid mineralization as an *in vitro* model system. A)** The small molecule dorsomorphin, a BMP inhibitor, induced mineral formation outside spheroids after 10 days of cell culture. For 2 μM dorsomorphin, spheroids were detectable, while for 10 μM of dorsomorphin cell death was present, as shown by the absence of F-actin staining. Spheroids were visualized through F-actin immunolabeling and hydroxyapatite deposition through the OsteoImage™ dye. **B)** SEM imaging revealed the presence of extracellular particles surrounding the spheroids. **C)** Immunolabeling of β-catenin and BMP2 allowed the visualization of these extracellular particles. **D)** WNT3a and WNT5 expression levels were increased in MSC spheroids compared to monolayer cultures (*P < 0.05). **E)** The WNT activator CHIR99021, combined with 4 mM Ca^2+^, induced mineralization in MSCs after 10 days of incubation. Mineralization was visualized through Alizarin Red staining.

Since spheroids are more representative of tissue physiology than monolayer cultures [12], we investigated if mineralized spheroids are useful as a model system to study the effects of small molecules. This is to demonstrate that this model has the potential to discover potential novel drug targets to inhibit or enhance mineralization events for regenerative medicine applications. Since our results demonstrated that BMP2 is highly upregulated in spheroid models compared to monolayer culture, we investigated the effect of dorsomorphin, a BMP inhibitor [42], on spheroid mineralization. After administering a combined treatment of Ca^2+^ and dorsomorphin to MSC spheroids for a 10-day period, we observed a marked increase in mineralization (Fig. 9A). At 2 μM dorsomorphin, spheroids were surrounded by mineral deposits, while the spheroids were visible by F-actin staining. At 10 μM dorsomorphin, no cells were detected as shown for F-actin immunolabeling, implicating cell death. It might be that inhibiting BMP signaling activates other pathways involved in mineralization, such as WNT signaling [52,53], or that in combination with the spheroid environment, toxic conditions are induced. Of interest, given the extensive mineralization observed outside the spheroids, it might be that particles emanating from the spheroids facilitated external mineralization events.

To further investigate the elevated increase of external mineralization, we applied SEM imaging on the spheroids cultured in basic medium, revealing particles outside the spheroids (Fig. 9B). These particles have a biological origin as supported by a positive immunolabeling of β-catenin and BMP2 (Fig. 9C). Here, β-catenin exhibited the most substantial presence in these particles, while a lower amount of BMP2 was also visible. Both β-catenin and BMP2 are present in extracellular vesicles and play essential roles in signal transduction [54,55]. However, more extensive research is needed to validate this claim. Potentially, these particles could contain complex protein compositions that can promote mineralization [56]. Next to these observations, tracking spheroid behavior over time using live cell imaging through bright-field revealed an accumulation of these particles (Supplementary Data File 5). These results suggest that the microwell-based spheroid mineralization model can also be used as a tool to study the biological processes involving extracellular-associated mineralization.

Previous research findings from our group demonstrated that an in-depth understanding of signaling events elicited by the biophysical context of a biomaterial can lead to the discovery of soluble cues that mimic such an environment [57]. Here, we wanted to demonstrate that this concept can also be applied to the biophysical context elicited by spheroids. Next to promoting BMP2 levels, a spheroid environment is known to elevate the expression of WNT ligands in MSCs [19]. Similarly, we validated this observation by measuring WNT3 and WNT5a expression levels in MSC spheroids after 20 days of culture in basic medium compared to an MSC monolayer culture (Fig. 9D). We wondered if this knowledge of the underlying pathways that promote osteogenesis in MSC spheroids can be applied in a monolayer culture. We therefore combined the WNT activator CHIR99021 [58] together with Ca^2+^ supplementation in an MSC monolayer culture in an attempt to mimic the beneficial effects of a spheroid environment (Fig. 9E). After 10 days of incubation in a monolayer culture with 4 mM Ca^2+^, mineralization was absent due to a suboptimal Ca^2+^ concentration. However, treating MSCs with a combination of 4 mM Ca^2+^ and 10 μM CHIR99021 resulted in highly pronounced mineralization, which is usually only achieved at higher Ca^2+^ concentrations (e.g., 8 mM Ca^2+^) or seen after a 21–28 day incubation with standard osteogenic medium. This observation demonstrates that the benefits of the spheroid environment can be mimicked in 2D as well, which is interesting for differentiation protocols without spheroid cultures.

These results illustrate that spheroid calcification models are useful tools for studying mineralization mechanisms and identifying small molecules influencing mineralization, which can be applied to a wide range of regenerative medicine applications.

## 3 Discussion

This study is the first to explore the effects of Ca^2+^, Pi, and DEX supplementation for achieving rapid mineralization in MSC spheroids. Previous research has demonstrated that the biophysical context of cellular aggregation primes MSCs towards an osteogenic lineage compared to a standard monolayer culture. This is attributed to altered cadherin, WNT, and BMP2 expression and signaling [8,18,19]. Similarly, we validated that a spheroid environment upregulates WNT3, WNT5a, and BMP2 while demonstrating that Ca^2+^ and Pi further enhance BMP2 expression. Similar effects were found for OPN and OCN in a time-dependent manner. Although BMP2 was highly upregulated in spheroid cultures exposed to either Ca^2+^ or Pi, only spheroids incubated with Ca^2+^ showed clear mineralization after 10 days. This implies that other mechanisms next to elevated BMP signaling are responsible for the observed mineralization. Interestingly, higher expression levels of RUNX2, considered an essential transcriptional factor in osteoblasts [35], were observed earlier in spheroids compared to monolayer cultures, with Ca, Pi or DEX supplementation not further improving expression levels. This provides strong evidence that the 3D environment promotes an osteogenic phenotype in MSCs.

Although the results demonstrate that Ca^2+^ and Pi supplementation in a spheroid environment promotes an osteogenic gene expression profile, it is unclear if the mineralization we observed is due to precipitation of minerals from the mineralization medium or biological mineralized deposition originating from the MSCs. Considering that concentrations higher than 4 mM Ca^2+^ induce precipitation without the presence of spheroids, and 4 mM Ca^2+^ induces a similar deposition after 20 days of cell culture, there is a strong indication that minerals originating from the culture medium play a role in spheroid mineralization. Nevertheless, our results suggest a strong biological effect in forming these minerals inside and outside the spheroids as shown by subjecting MSCs from different donors to calcification media. Furthermore, mineralization in the Pi condition was clearly affected in the presence of DEX. Gene expression analysis demonstrated that DEX negatively influenced BMP2 and OPN expression while promoting ALP levels. Since ALP is an essential contributor to mineralization [50], these higher ALP levels might promote mineralization observed in the 2 mM Pi condition supplemented with DEX. Similarly, the addition of the small molecule dorsomorphin significantly increased mineralized deposition outside the spheroids. Nevertheless, even without a biological component to induce mineralization, the mineralization formed in the culture medium might be used as a convenient (micro)scaffolding inside spheroids, which is not easily realized by other means. Additional research will be required to elucidate the exact mechanism of action of mineralization formation in our experimental setups.

Our results highlight that optimizing Ca^2+^ and Pi concentrations is essential to avoid toxic conditions while maintaining an experimental setup that allows fast spheroid mineralization. This is also an important consideration for experimental setups that use decreased fetal bovine serum (FBS) concentrations or serum-free defined conditions, since FBS contains an unneglectable amount of Ca^2+^ and proteins that inhibit Ca^2+^-associated toxicity [59]. On the other hand, FBS also contains oxalate that, together with an increased presence of Ca^2+^, forms calcium oxalate, of which we do not know what their implications are in our spheroid setup. Besides variations in cell culture medium or serum, the cell source is a crucial factor to consider for generating mineralized spheroids, as different MSC sources are shown to vary in their osteogenic differentiation and mineralization potential [60]. Additionally, MSC donor variability significantly impacted the rate of calcification in spheroids, a phenomenon that was also shown previously in 2D MSC cultures [30]. Consequently, it is essential to refine incubation durations and adjust Ca^2+^ or Pi concentrations to determine the ideal culture conditions for different MSC sources/donors. The experimental setup itself to generate spheroids could influence Ca^2+^ and Pi-induced toxicity. For example, in our study, the microwells for generating spheroids had the disadvantage of accumulating mineralized deposits at high Ca^2+^ and Pi concentrations. This phenomenon might have contributed to elevated toxicity levels. While the mitochondrial structure appeared intact in spheroids exposed to calcification, it is possible that upon reaching a specific calcification threshold, mitochondrial stress from Ca^2+^ or Pi ions is triggered, resulting in cell death [61]. To better understand the precise mechanisms behind the observed cell death, further studies are necessary, which will aid in designing more refined experimental setups. Regarding this, using systems based on microfluidics and microporous microwells, in which spheroids are subjected to continuous medium replenishment [[62], [63], [64]], might allow even higher Ca^2+^ and Pi concentrations to induce faster mineralization while avoiding toxicity.

Our model more resembles certain aspects of tissue physiology compared to traditional 2D culture setups. Nevertheless, additional improvements can be made if the goal is to mimic bone physiology. Notably, our model utilized only one cell type, whereas bone is composed of multiple cell types, including osteoblasts, osteocytes, and osteoclasts. In the future, it would be interesting to develop co-culture experiments where differentiated MSC spheroids are incubated together with osteoclasts to investigate if osteoclasts can degrade the calcification inside spheroids. Furthermore, although we did indicate the presence of hydroxyapatite surrounding the cells in the spheroids, we did not characterize the matrix composition or collagen architecture. In the future, examining this will be crucial to assess bone tissue similarities. In case of immature matrix development, including matrix compounds in the spheroids might further refine the model.

Nevertheless, we envision that Ca^2+^ or Pi supplementation in spheroid culture systems, an easy-to-use protocol, is applicable in various contexts. First, the mineralized MSC spheroids from this study can be useful for bone tissue engineering applications. For example, another study demonstrated that human-periosteum-derived spheroids could be assembled in scaffold-free constructs to heal long bone defects [13]. Follow-up studies should investigate if mineralized MSC spheroids are suitable for this approach as well. Next to MSC spheroids, Ca^2+^ or Pi supplementation would be interesting for enhancing iPSCs differentiation protocols towards the osteogenic lineage, especially for lowering the length of differentiation protocols, considering that standard osteogenic compounds such as β-glycerophosphate and DEX are still applied to differentiate iPSCs towards osteoblast lineage [65]. Besides focusing on bone regeneration, we believe that the rapid mineralization of spheroids can also be applied in disease models involving unwanted calcification of tissues to facilitate screening experiments. Vascular calcification in atherosclerosis, calcific tendinitis, fibrodysplasia ossificans progressive, and primary familial brain calcification are all examples of ectopic calcification leading to different disease pathologies [[66], [67], [68], [69]]. Since drug screens are often applied to spheroid or organoid models to elucidate pathway mechanism of action or to find novel therapeutics [70,71], we demonstrated as a proof-of-concept that small molecules such as dorsomorphin applied on mineralized spheroids lead to phenomena that influences calcification. In this regard, it will also be important to determine if the observed extracellular particles are microvesicles, exosomes, or apoptotic bodies and if they play a role in calcification events. To conclude, we envision that these research results will inspire the development of innovative mineralization models for regenerative medicine applications.

## 4 Materials and methods

### 4.1 Microwell array fabrication

First, a brass mold comprising an array of 289 microcavities in the form of circular-cylindrical dead-end holes, each with a diameter of 550 μm and a depth of 800 μm, was fabricated by microdrilling. Next, through microthermoforming [72], microwells were fabricated onto 50 μm-thin PC films. Detailed procedures of how to generate the microwells can be found elsewhere [35]. The cavities formed were approximately 500 μm in diameter and 300 μm in depth, which were verified through a confocal laser scanning microscope (Keyence 3D Laser Scanning Microscope VK-X250K coupled with Keyence MultiFileAnalyzer software). Microwell arrays were cropped with a surgical knife to fit the wells in a 24- or 96-well cell culture plates. After insertion into either a 24-well or a 96-well plate, an O-ring (ERIKS) was subsequently inserted to secure the position of the microwell array. 70 % ethanol was used to sterilize the microwells, after which they were washed 3x with phosphate-buffered saline (PBS). Afterward, microwells were treated with 1 % Pluronic™ F-108 (Sigma-Aldrich) for 24 h at 37 °C to create a low-binding coating on the surface of the microwells to prevent the cells from adhering. After 3x washing with PBS, microwells were ready for cell culture experiments.

### 4.2 Cell culture

Bone marrow-derived human mesenchymal stromal cells (BM-hMSCs) were isolated from a human bone marrow aspirate of which informed consent was given. The studies involving human participants were reviewed and approved by the Medical Ethical Committee (METC) of the Maastricht University Medical Center (#15-4-274). Basic culture medium for BM-hMSCs consisted of αMEM GlutaMAX™, no nucleosides (Gibco) and was supplemented with 10 % v/v fetal bovine serum (FBS; Sigma-Aldrich), 0.2 mM ascorbic-acid-2-phosphate (ASAP; Thermo Fisher), and 100 U/mL Penicillin/Streptomycin. Cells were grown in tissue culture flasks (Nunc) while in a humid atmosphere at 37 °C and 5 % CO_2_. For experiments involving 2D culture, BM-hMSCs were trypsinized through trypsin (Gibco), and seeded at 5000 cells/cm^2^ in 24-well plate format. For experiments involving microwell arrays, BM-hMSCs were trypsinized through trypsin (Gibco) and seeded at passage 4 in the microwells at a density of 20,000 cells/cm^2^ in either 24-well or 96-well plate format.

For standard osteogenic differentiation, basic medium was supplemented with 100 nM dexamethasone (Sigma-Aldrich) and 10 mM β-glycerol phosphate (Sigma-Aldrich). To obtain media containing Ca^2+^ and PO_4_^3−^, respectively 0.8 M CaCl_2_ or 0.8 M Na_2_HPO_4_·2H_2_O were dissolved in 5 ml 25 mM Hepes, after which the pH was adjusted to 7.4. A stock solution was obtained by subsequently dissolving the buffer in 500 ml basic media to a final concentration of 8 mM CaCl_2_ or 8 mM Na_2_HPO_4_·2H_2_O. Further dilutions were prepared by mixing the 8 mM stock with basic media to obtain final concentrations of 2, 4, 6 mM.

### 4.3 Scanning electron microscopy

Spheroids were fixed with 4 v/v% paraformaldehyde in phosphate-buffered saline (PBS) for 30 min and dehydrated in a graded series of ethanol (at room temperature): 30 % for 30 min (twice), 50 % for 30 min, 70%–80%–90%–96 % for 10 min each, and finally 100 % for 10 min (thrice). Samples were dried overnight using hexamethyldisilazane (Sigma-Aldrich) at room temperature and coated with a thin layer of gold (10 nm) in a sputter coater (Cressington 108). For imaging, an FEI/Philips XL30 was applied.

### 4.4 Focused ion beam scanning electron microscopy

Spheroids were fixed after 10 days in culture and prepared for 3D electron microscopy. Initially, samples were fixed with 2.5 % glutaraldehyde (Merck) in 0.1 m cacodylic acid sodium salt trihydrate (cacodylate buffer; ACROS Organics) for 24 h at 4 °C. Subsequently, samples were washed with 0.1 m cacodylate buffer and postfixed with 1 % osmium tetroxide (Electron Microscopy Sciences) in the same buffer containing 1.5 % potassium ferricyanide [K_3_Fe(CN)_6_] (Merck) for 1 h in darkness at 4 °C. Then, spheroids were dehydrated in a graded series of ethanol (70%–90%–100 %) (Merck), with each step repeated twice for 30 min. Samples were infiltrated with Epon resin (LADD) for 2 days, embedded in the same resin, and polymerized at 60 °C for 48 h. After fixation, the Epon blocks were trimmed down to the aggregates using a UC6 ultramicrotome (Leica) and diamond trimming knife (Diatome). Blocks were glued onto SEM stubs using superglue, coated with a carbon layer, and embedded into silver paint to prevent charging. Samples were then transferred into the Scios DualBeam SEM (ThermoFisher Scientific) and further processed for milling using Slice and View version 3.0. The process started with a FIB that milled a nanometer-thin layer from the sample, and subsequently, each freshly produced surface was imaged with SEM [[56], [57], [58]]. These steps were consecutively repeated until the whole 3D object was ablated and imaged. Regular cross sections were milled at 30 kV and 1 nA beam current. Within each step, FIB removed 20 nm of the Epon blocks (containing spheroids), and the fresh layers were imaged with SEM with an acceleration voltage of 2 keV. Dwell time was 300 ns per frame, and volumetric range image integration (32 images) was performed. The pixel size was 10 × 10 nm^2,^ and the image dimensions were 6144 (width) and 4096 (height) in pixels. The individual FIB-SEM slices were aligned using the DipImage Matlab image processing toolkit (http://www.diplib.org/). 3D figures were rendered using Amira 6.5.0 (Thermo-Scientific).

For higher resolution TEM analysis, 60 nm ultrathin sections were cut using a diamond knife (Diatome) on a Leica UC7 ultramicrotome, and transferred onto 50 Mesh copper grids covered with a formvar and carbon film. Sections were post-stained with uranyl acetate and lead citrate. All TEM data were collected autonomously as virtual nanoscopy slides [73] on FEI Tecnai T12 microscopes at 120 kV using an Eagle camera. Data were stitched, uploaded, shared and annotated using Omero (https://www.openmicroscopy.org/omero/) and PathViewer (Glencoe Software, Inc.).

### 4.5 Fourier-transform infrared spectroscopy

For FTIR analysis preparation, we fixed the spheroids with 4 % PFA, then flushed them from the microwell arrays using PBS, pooling eight wells from a 96-well plate per condition. After pooling, the spheroids were centrifuged at 300 g for 5 min. Post centrifugation, we removed the PBS and dehydrated the spheroids using a graded ethanol series (70 %, 90 %, 100 %), with each condition applied twice for 30 min. The spheroids were then dried overnight with hexamethyldisilazane (Sigma-Aldrich) at room temperature. ATR-FTIR spectra were captured using a Nicolet iS50 FT-IR instrument (ThermoFisher) in the 4000–400 cm^−1^ range. 16 sample scans and 16 background scans were recorded.

### 4.6 Immunocytochemistry

After cell culture, cells were washed with PBS (Merck) and fixed with 4 % (w∕v) paraformaldehyde (Sigma-Aldrich) for 5 min at 37 °C. After a washing step, permeabilization was achieved with 0.1 % (v/v) Triton X-100 (Acros Organics) and blocked with goat serum (1:100; Sigma-Aldrich) in PBT (PBS, 0.02 % Triton-X-100, 0.5 % bovine serum albumin (BSA); VWR) for 1 h. Afterward, cells were incubated with the primary antibody in PBT for 1 h. After a washing step, cells were incubated with a secondary antibody conjugated to an Alexa Fluor (4 μg/ml; ThermoFisher) and Phalloidin conjugated to an Alexa Fluor (4 μg/ml; ThermoFisher) in PBT for 1 h. The nucleus was counterstained with Hoechst33258 (5 μg/ml; Sigma-Aldrich) for 10 min. After labeling the nuclei and F-actin through Hoechst 33,258 and Phalloidin, respectively, labeling of calcium phosphate minerals in spheroid cultures was achieved using an OsteoImage™ Mineralization Assay (Lonza) as per manufacturer's instructions. After a subsequent washing step, microwell arrays were mounted on glass bottom well plates with mounting media (Dako) for confocal imaging. For epifluorescent imaging, microwell arrays remained in the 96-well plate. Washing steps were performed in triplicate with PBT. Primary antibodies used in this study are anti-BMP2 antibody (2.5 μg/ml; Abcam; ab6285), anti-β-catenin antibody (2.5 μg/ml; Abcam; ab2365). Listed antibody concentrations are their final dilutions.

To assess mineralization in 2D cultures, cells were fixed overnight at 4 °C with 4 % formaldehyde (VWR) in PBS. Mineralized deposits were subsequently stained with a 2 % Alizarin Red S solution (pH = 4.2) for 30 min. Excess staining was washed off with demineralized water.

### 4.7 Image analysis

Epifluorescent images were acquired through a fully automated Nikon Eclipse Ti–U microscope in combination with an Andor Zyla 5.5 4 MP camera. To obtain confocal images, a Leica TCS confocal laser scanning microscopy was used. The spheroid size was calculated by analyzing fluorescent images through CellProfiler version 4.0.5 [74], applying custom-made pipelines. Briefly, after illumination corrections, the spheroid morphology was captured by Otsu adaptive thresholding method applied on the F-actin image channel. Spheroids touching the border of the image were filtered out of the dataset. The imaging software Fiji was used for other image processing and visualization [75].

### 4.8 Quantitative PCR

Total RNA was isolated by using the RNeasy Mini Kit (Qiagen) according to the manufacturer's protocol. Reverse transcription on RNA samples was achieved using an iScriptTM cDNA synthesis kit (Bio-Rad). Quantitative PCR (RT-qPCR) on cDNA samples was performed using the iQ SYBR Green Supermix (Bio-Rad) in a CFX96TM Real-Time PCR Detection Kit (Bio-Rad). Glyceraldehyde 3-phosphate dehydrogenase (GAPDH) and TATA-Box Binding Protein (TBP) were used as housekeeping genes and relative expression was determined using the ΔΔCt method through the qBase + software. Primer sequences are listed in Supplementary Data File 6.

### 4.9 Statistical analysis

Statistical analyses was performed through Prism version 9.3.1 (GraphPad, San Diego, USA). For the assessment of image and qPCR analysis data, a one-way ANOVA was utilized to assess significant variations among different experimental conditions. Significance was attributed to observations when the P-value was less than 0.05. qPCR data was derived from a minimum of three independent experiments.

## CRediT authorship contribution statement

**Steven Vermeulen:** Conceptualization, Methodology, Software, Validation, Formal analysis, Investigation, Writing – original draft, Visualization. **Kèvin Knoops:** Methodology, Software, Investigation, Resources, Data curation, Formal analysis. **Hans Duimel:** Investigation. **Maryam Parvizifard:** Investigation. **Denis van Beurden:** Investigation. **Carmen López Iglesias:** Formal analysis, Conceptualization, Resources. **Stefan Giselbrecht:** Conceptualization, Resources. **Roman Truckenmüller:** Conceptualization, Resources, Writing – review & editing. **Pamela Habibović:** Conceptualization, Writing – review & editing, Supervision, Funding acquisition. **Zeinab Tahmasebi Birgani:** Conceptualization, Writing – review & editing, Validation, Investigation, Supervision, Funding acquisition, Project administration.

## Declaration of competing interest

The authors declare the following financial interests/personal relationships which may be considered as potential competing interests:Stefan Giselbrecht reports a relationship with 300MICRONS GmbH that includes: board membership. Roman Truckenmuller reports a relationship with 300MICRONS GmbH that includes: board membership.

## Data Availability

The data supporting the findings of this study are openly available in DataverseNL at https://doi.org/10.34894/T4TPLJ.

## Appendix A Supplementary data

Multimedia component 1 Multimedia component 1

Multimedia component 2 **Supplementary Data File 1:** Initial moments of MSC spheroid formation. Multimedia component 2

Multimedia component 3 **Supplementary Data File 2:** FIB-SEM imaging of an MSC spheroid cultured in basic conditions for 10 days. Multimedia component 3

Multimedia component 4 **Supplementary Data File 3:** FIB-SEM imaging of an MSC spheroid cultured with 4 mM Ca^2+^ for 10 days. Multimedia component 4

Multimedia component 5 **Supplementary Data File 4:** FIB-SEM imaging of an MSC spheroid cultured with 2 mM Pi + DEX for 10 days. Multimedia component 5

Multimedia component 6 **Supplementary Data File 5: Extracellular particle formation and accumulation from MSC spheroids visualized through bright-field imaging**. The blue arrow embedded in the movie indicates extracellular particle formation. Each frame represents 10 min culture time. Multimedia component 6

## References

[1] Woolf A., D, Pfleger B. (2003). Burden of major musculoskeletal conditions. Bull. World Health Organ..

[2] Evans J.T., Evans J.P., Walker R.W., Blom A.W., Whitehouse M.R., Sayers A. (2019). How long does a hip replacement last? A systematic review and meta-analysis of case series and national registry reports with more than 15 years of follow-up. Lancet (London, England).

[3] Hao C.P., Cao N.J., Zhu Y.H., Wang W. (2021). The osseointegration and stability of dental implants with different surface treatments in animal models: a network meta-analysis. Sci. Rep..

[4] Dimitriou R., Elena J., McGonagle D., Giannoudis P. (2011). Bone regeneration: current concepts and future directions. BMC Med..

[5] Pape H.C., Evans A., Kobbe P. (2010). Autologous bone graft: properties and techniques. J. Orthop. Trauma.

[6] Tonk G., Yadav P.K., Agarwal S., Jamoh K. (2022). Donor site morbidity in autologous bone grafting – a comparison between different techniques of anterior iliac crest bone harvesting: a prospective study. J. Orthop. Trauma Rehabil..

[7] Clevers H. (2016). Modeling development and disease with organoids. Cell.

[8] Passanha F.R., Geuens T., Konig S., Van Blitterswijk C.A., Lapointe V.L.S. (2020). Cell culture dimensionality influences mesenchymal stem cell fate through cadherin-2 and cadherin-11. Biomaterials.

[9] Gallo J., Sutherland R.M., Murphy B.J., Kramer R.H. (1994). Selective down-regulation of integrin receptors in spheroids of squamous cell carcinoma. Cancer Res..

[10] Tsai A.C., Liu Y., Yuan X., Ma T. (2015). Compaction, fusion, and functional activation of three-dimensional human mesenchymal stem cell aggregate. Tissue Eng..

[11] Huch M., Koo B.K. (2015). Modeling mouse and human development using organoid cultures. Devenir.

[12] Dutta D., Heo I., Clevers H. (2017). Disease modeling in stem cell-derived 3D organoid systems. Trends Mol. Med..

[13] Nilsson Hall G., Mendes L.F., Gklava C., Geris L., Luyten F.P., Papantoniou I. (2020). Developmentally engineered callus organoid bioassemblies exhibit predictive in vivo long bone healing. Adv. Sci..

[14] Dominici M., Le Blanc K., Mueller I., Slaper-Cortenbach I., Marini F.C., Krause D.S., Deans R.J., Keating A., Prockop D.J., Horwitz E.M. (2006). Minimal criteria for defining multipotent mesenchymal stromal cells. The International Society for Cellular Therapy position statement. Cytotherapy.

[15] Solchaga L.A., Penick K.J., Welter J.F. (2011).

[16] Yamamoto M., Kawashima N., Takashino N. (2013). Three-dimensional spheroid culture promotes odonto/osteoblastic differentiation of dental pulp cells, Arch. Oral Biol.

[17] Yeh H.Y., Liu B.H., Sieber M., hui Hsu S. (2014). Substrate-dependent gene regulation of self-assembled human MSC spheroids on chitosan membranes. BMC Genom..

[18] Kabiri M., Kul B., Lott W.B., Futrega K., Ghanavi P., Upton Z., Doran M.R. (2012). 3D mesenchymal stem/stromal cell osteogenesis and autocrine signalling. Biochem. Biophys. Res. Commun..

[19] hui Hsu S., Huang G.S. (2013). Substrate-dependent Wnt signaling in MSC differentiation within biomaterial-derived 3D spheroids. Biomaterials.

[20] Xu L., Meng F., Ni M., Lee W.Y.W., Li G. (2013). N-cadherin regulates osteogenesis and migration of bone marrow-derived mesenchymal stem cells. Mol. Biol. Rep..

[21] Lin G.L., Hankenson K.D. (2011). Integration of BMP, Wnt, and notch signaling pathways in osteoblast differentiation. J. Cell. Biochem..

[22] Vermeulen S., Birgani Z.T., Habibovic P. (2022). Biomaterial-induced pathway modulation for bone regeneration. Biomaterials.

[23] Quelch K.J., Melick R.A., Bingham P.J., Mercuri S.M. (1983). Chemical composition of human bone. Arch. Oral Biol..

[24] Veldurthy V., Wei R., Oz L., Dhawan P., Jeon Y.H., Christakos S. (2016). Vitamin D, calcium homeostasis and aging. Bone Res.

[25] Penido M.G.M.G., Alon U.S. (2012). Phosphate homeostasis and its role in bone health. Pediatr. Nephrol..

[26] Barradas A.M.C., Fernandes H.A.M., Groen N., Chai Y.C., Schrooten J., Van de Peppel J., Van Leeuwen J.P.T.M., Van Blitterswijk C.A., De Boer J. (2012). A calcium-induced signaling cascade leading to osteogenic differentiation of human bone marrow-derived mesenchymal stromal cells. Biomaterials.

[27] Choudhary S., Kumar A., Kale R.K., Raisz L.G., Pilbeam C.C. (2004). Extracellular calcium induces COX-2 in osteoblasts via a PKA pathway. Biochem. Biophys. Res. Commun..

[28] Tada H., Nemoto E., Foster B.L., Somerman M.J., Shimauchi H. (2011). Phosphate increases bone morphogenetic protein-2 expression through cAMP-dependent protein kinase and ERK1/2 pathways in human dental pulp cells. Bone.

[29] Beck G.R., Knecht N. (2003). Osteopontin regulation by inorganic phosphate is ERK1/2-, protein kinase C-, and proteasome-dependent. J. Biol. Chem..

[30] Danoux C.B.S.S., Bassett D.C., Othman Z., Rodrigues A.I., Reis R.L., Barralet J.E., Van Blitterswijk C.A., Habibovic P. (2015). Elucidating the individual effects of calcium and phosphate ions on hMSCs by using composite materials. Acta Biomater..

[31] Hamidouche Z., Haÿ E., Vaudin P., Charbord P., Schüle R., Marie P.J., Fromigué O. (2008). FHL2 mediates dexamethasone‐induced mesenchymal cell differentiation into osteoblasts by activating Wnt/β‐catenin signaling‐dependent Runx2 expression. Faseb. J..

[32] Sato N., Aoyama Y., Yamanaka J., Toyotama A., Okuzono T. (2017). Particle adsorption on hydrogel surfaces in aqueous media due to van der Waals attraction. Sci. Rep..

[33] Kakni P., Hueber R., Knoops K., López-Iglesias C., Truckenmüller R., Habibovic P., Giselbrecht S. (2020). Intestinal organoid culture in polymer film-based microwell arrays. Adv. Biosyst..

[34] Samal P., Maurer P., van Blitterswijk C., Truckenmüller R., Giselbrecht S. (2020). A new microengineered platform for 4D tracking of single cells in a stem-cell-based in vitro morphogenesis model. Adv. Mater..

[35] Fois M.G., Tahmasebi Birgani Z.N., Guttenplan A.P.M., van Blitterswijk C.A., Giselbrecht S., Habibović P., Truckenmüller R.K. (2022). Assessment of cell–material interactions in three dimensions through dispersed coaggregation of microsized biomaterials into tissue spheroids. Small.

[36] An S., Gao Y., Huang Y., Jiang X., Ma K., Ling J. (2015). Short-term effects of calcium ions on the apoptosis and onset of mineralization of human dental pulp cells in vitro and in vivo. Int. J. Mol. Med..

[37] Meleti Z., Shapiro I.M., Adams C.S. (2000). Inorganic phosphate induces apoptosis of osteoblast-like cells in culture. Bone.

[38] Langenbach F., Berr K., Naujoks C., Hassel A., Hentschel M., Depprich R., Kubler N.R., Meyer U., Wiesmann H.P., Kögler G., Handschel J. (2011). Generation and differentiation of microtissues from multipotent precursor cells for use in tissue engineering. Nat. Protoc..

[39] Jiang B., Yan L., Miao Z., Li E., Wong K.H., Xu R.H. (2017). Spheroidal formation preserves human stem cells for prolonged time under ambient conditions for facile storage and transportation. Biomaterials.

[40] Jeger-Madiot N., Arakelian L., Setterblad N., Bruneval P., Hoyos M., Larghero J., Aider J.L. (2021). Self-organization and culture of Mesenchymal Stem Cell spheroids in acoustic levitation. Sci. Rep..

[41] Youle R.J., Van Der Bliek A.M. (2012). Mitochondrial fission, fusion, and stress. Science.

[42] Pedraza C.E., Chien Y.C., McKee M.D. (2008). Calcium oxalate crystals in fetal bovine serum: implications for cell culture, phagocytosis and biomineralization studies in vitro. J. Cell. Biochem..

[43] Daudon M., Jungers P., Bazin D. (2008). Stone morphology: implication for pathogenesis. AIP Conf. Proc..

[44] Belliveau J., Griffin H. (2001). The solubility of calcium oxalate in tissue culture media. Anal. Biochem..

[45] Bruderer M., Richards R.G., Alini M., Stoddart M.J. (2014). Role and regulation of runx2 in osteogenesis. Eur. Cell. Mater..

[46] Wu M., Chen G., Li Y.P. (2016). TGF-β and BMP signaling in osteoblast, skeletal development, and bone formation, homeostasis and disease. Bone Res.

[47] Franzen A., Heinegard D. (1985). Isolation and characterization of two sialoproteins present only in bone calcified matrix. Biochem. J..

[48] Zoch M.L., Clemens T.L., Riddle R.C. (2016). New insights into the biology of osteocalcin. Bone.

[49] Rammelt S., Neumann M., Hanisch U., Reinstorf A., Pompe W., Zwipp H., Biewener A. (2005). Osteocalcin enhances bone remodeling around hydroxyapatite/collagen composites. J. Biomed. Mater. Res., Part A.

[50] Golub E.E., Boesze-Battaglia K. (2007). The role of alkaline phosphatase in mineralization. Curr. Opin. Orthop..

[51] Holm E., Gleberzon J.S., Liao Y., Sørensen E.S., Beier F., Hunter G.K., Goldberg H.A. (2014). Osteopontin mediates mineralization and not osteogenic cell development in vitro. Biochem. J..

[52] He X.C., Zhang J., Tong W.G., Tawfik O., Ross J., Scoville D.H., Tian Q., Zeng X., He X., Wiedemann L.M., Mishina Y., Li L. (2004). BMP signaling inhibits intestinal stem cell self-renewal through suppression of Wnt-β-catenin signaling. Nat. Genet..

[53] Voorneveld P.W., Kodach L.L., Jacobs R.J., Van Noesel C.J.M., Peppelenbosch M.P., Korkmaz K.S., Molendijk I., Dekker E., Morreau H., Van Pelt G.W., Tollenaar R.A.E.M., Mesker W., Hawinkels L.J.A.C., Paauwe M., Verspaget H.W., Geraets D.T., Hommes D.W., Offerhaus G.J.A., Van Den Brink G.R., Ten Dijke P., Hardwick J.C.H. (2015). The BMP pathway either enhances or inhibits the Wnt pathway depending on the SMAD4 and p53 status in CRC. Br. J. Cancer.

[54] Nahar N.N., Missana L.R., Garimella R., Tague S.E., Anderson H.C. (2008). Matrix vesicles are carriers of bone morphogenetic proteins (BMPs), vascular endothelial growth factor (VEGF), and noncollagenous matrix proteins. J. Bone Miner. Metabol..

[55] Bussche L., Rauner G., Antonyak M., Syracuse B., McDowell M., Brown A.M.C., Cerione R.A., Van De Walle G.R. (2016). Microvesicle-mediated wnt/β-catenin signaling promotes interspecies mammary stem/progenitor cell growth. J. Biol. Chem..

[56] Golub E.E. (2009). Role of matrix vesicles in biomineralization. Biochim. Biophys. Acta.

[57] Vermeulen S., Roumans N., Honig F., Carlier A., Hebels D., Dede Eren A., ten Dijke P., Vasilevich A., de Boer J. (2020). Mechanotransduction is a context-dependent activator of TGF-β signaling in mesenchymal stem cells. Biomaterials.

[58] Bain J., Plater L., Elliott M., Shpiro N., Hastie C.J., Mclauchlan H., Klevernic I., Arthur J.S.C., Alessi D.R., Cohen P. (2007). The selectivity of protein kinase inhibitors: a further update. Biochem. J..

[59] Blankenship J.R., Heitman J. (2005). Calcineurin is required for Candida albicans to survive calcium stress in serum. Infect. Immun..

[60] Mohamed-Ahmed S., Fristad I., Lie S.A., Suliman S., Mustafa K., Vindenes H., Idris S.B. (2018). Adipose-derived and bone marrow mesenchymal stem cells: a donor-matched comparison. Stem Cell Res. Ther..

[61] Duchen M.R. (2000). Mitochondria and calcium: from cell signalling to cell death. J. Physiol..

[62] Vadivelu R.K., Kamble H., Shiddiky M.J.A., Nguyen N.T. (2017). Microfluidic technology for the generation of cell spheroids and their applications. Micromachines.

[63] Giselbrecht S., Gietzelt T., Gottwald E., Trautmann C., Truckenmüller R., Weibezahn K.F., Welle A. (2006). 3D tissue culture substrates produced by microthermoforming of pre-processed polymer films. Biomed. Microdevices.

[64] Baptista D., Moreira Teixeira L., Barata D., Tahmasebi Birgani Z., King J., Van Riet S., Pasman T., Poot A.A., Stamatialis D., Rottier R.J., Hiemstra P.S., Carlier A., Van Blitterswijk C., Habibović P., Giselbrecht S., Truckenmüller R. (2022). 3D lung-on-chip model based on biomimetically microcurved culture membranes. ACS Biomater. Sci. Eng..

[65] Zujur D., Kanke K., Lichtler A.C., Hojo H., Il Chung U., Ohba S. (2017). Three-dimensional system enabling the maintenance and directed differentiation of pluripotent stem cells under defined conditions. Sci. Adv..

[66] Ross R. (1999). Atherosclerosis - an inflammatory disease. Mech. Dis..

[67] Darrieutort-Laffite C., Blanchard F., Le Goff B. (2018). Calcific tendonitis of the rotator cuff: from formation to resorption. Jt. Bone Spine..

[68] Mahboubi S., Glaser D.L., Shore E.M., Kaplan F.S. (2001). Fibrodysplasia ossificans progressiva. Pediatr. Radiol..

[69] Keller A., Westenberger A., Sobrido M.J., García-Murias M., Domingo A., Sears R.L., Lemos R.R., Ordoñez-Ugalde A., Nicolas G., Da Cunha J.E.G., Rushing E.J., Hugelshofer M., Wurnig M.C., Kaech A., Reimann R., Lohmann K., Dobričić V., Carracedo A., Petrović I., Miyasaki J.M., Abakumova I., Mäe M.A., Raschperger E., Zatz M., Zschiedrich K., Klepper J., Spiteri E., Prieto J.M., Navas I., Preuss M., Dering C., Janković M., Paucar M., Svenningsson P., Saliminejad K., Khorshid H.R.K., Novaković I., Aguzzi A., Boss A., Le Ber I., Defer G., Hannequin D., Kostić V.S., Campion D., Geschwind D.H., Coppola G., Betsholtz C., Klein C., Oliveira J.R.M. (2013). Mutations in the gene encoding PDGF-B cause brain calcifications in humans and mice. Nat. Genet..

[70] Friedrich J., Seidel C., Ebner R., Kunz-Schughart L.A. (2009). Spheroid-based drug screen: considerations and practical approach. Nat. Protoc..

[71] Driehuis E., Kretzschmar K., Clevers H. (2020). Establishment of patient-derived cancer organoids for drug-screening applications. Nat. Protoc..

[72] Truckenmüller R., Giselbrecht S., Rivron N., Gottwald E., Saile V., Van Den Berg A., Wessling M., Van Blitterswijk C. (2011). Thermoforming of film-based biomedical microdevices. Adv. Mater..

[73] Faas F.G.A., Cristina Avramut M., van den Berg B.M., Mieke Mommaas A., Koster A.J., Ravelli R.B.G. (2012). Virtual nanoscopy: generation of ultra-large high resolution electron microscopy maps. J. Cell Biol..

[74] McQuin C., Goodman A., Chernyshev V., Kamentsky L., Cimini B.A., Karhohs K.W., Doan M., Ding L., Rafelski S.M., Thirstrup D., Wiegraebe W., Singh S., Becker T., Caicedo J.C., Carpenter A.E. (2018). CellProfiler 3.0: next-generation image processing for biology. PLoS Biol..

[75] Schindelin J., Arganda-Carrera I., Frise E., Verena K., Mark L., Tobias P., Stephan P., Curtis R., Stephan S., Benjamin S., Jean-Yves T., Daniel J.W., Volker H., Kevin E., Pavel T., Albert C. (2009). Fiji - an Open platform for biological image analysis. Nat. Methods.

