# Supplementary material for: An in vitro model system based on calcium- and phosphate ion-induced hMSC spheroid mineralization
Source: Mater Today Bio. 2023 Nov 7;23:100844. doi: 10.1016/j.mtbio.2023.100844 (PMC10682137; doi:10.1016/j.mtbio.2023.100844)
Supplement: Multimedia component 1 [file mmc1.docx]

| ***Homo Sapiens*** | |
| --- | --- |
| **Primer Name** | **Sequence** |
| *COL-I-F* | GGCTCCTGCTCCTCTTAGCG |
| *COL-I-R* | CATGGTACCTGAGGCCGTTC |
| *BMP2-F* | CGAAATTCCCCGTGAC |
| *BMP2-R* | AGTTACTAGCAATGGCCT |
| *OCN-F* | GGCAGCGAGGTAGTGAAGAG |
| *OCN-R* | GATGTGGTCAGCCAACTCGT |
| *OPN-F* | CCAAGTAAGTCCAACGAAAG |
| *OPN-R* | GGTGATGTCCTCGTCTGTA |
| *RUNX2-F* | TCCGGAATGCCTCTGCTGTTATGA |
| *RUNX2-R* | AAGGTGAAACTCTTGCCTCGTCCA |
| *ALP-F* | ACAAGCACTCCCACTTCATCTGGA |
| *ALP-R* | TCACGTTGTTCCTGTTCAGCTCGT |
| *WNT3-F* | AGGGCACCTCCACCATTTG |
| *WNT3-R* | GACACTAACACGCCGAAGTCA |
| *WNT5a-F* | ATTCTTGGTGGTCGCTAGGTA |
| *WNT5a-R* | CGCCTTCTCCGATGTACTGC |
| *GAPDH-F* | GGCTGAGAACGGGAAGCTTGTCAT |
| *GAPDH-R* | CAGCCTTCTCCATGGTGGTGAAGA |
| *TBP-F* | GAGCTGTGATGTGAAGTTTCC |
| *TBP-R* | TCTGGGTTTGATCATTCTGTAG |
